# Apolipoprotein L3 enhances CD8+ T cell antitumor immunity of colorectal cancer by promoting LDHA-mediated ferroptosis

**DOI:** 10.7150/ijbs.74985

**Published:** 2023-02-13

**Authors:** Yang Lv, WenTao Tang, YuQiu Xu, WenJu Chang, ZhiYuan Zhang, Qi Lin, MeiLing Ji, QingYang Feng, GuoDong He, JianMin Xu

**Affiliations:** 1 Department of General Surgery, Zhongshan Hospital, Fudan University, Shanghai, China; 2 Cancer Center, Zhongshan Hospital, Fudan University, Shanghai, China; 3 Shanghai Engineering Research Center of Colorectal Cancer Minimally Invasive Surgery, Shanghai, China

**Keywords:** colorectal cancer, ferroptosis, CD8+ T cell infiltration, ubiquitin

## Abstract

**Aim:** Colorectal cancer (CRC) is the leading cause of cancer associated death worldwide and immune checkpoint blockade therapy only benefit a small set of CRC patients. Tumor ferroptosis of CRC reflected immune-activation in our previous findings. Understanding the mechanisms underlying how to bolster CD8+ T cells function through ferroptosis in CRC tumor microenvironment (TME) will greatly benefit cancer immunotherapy.

**Methods:** Genes between ferroptosis and CD8+ T cell function in CRC were screened through Cox, WGCNA and differential expression analysis. Immunohistochemistry and Immunofluorescence analysis were performed. Co-immunoprecipitation were performed to determine protein-protein interaction, mRNA level was determined by qRT-PCR. RSL3 was used to induce ferroptosis, and ferroptosis levels were evaluated by measuring Transmission Electron Microscope analysis, MDA, Fe^2+^level and cell viability.

**Results:** We screened APOL3 as the significant modulator for ferroptosis-related CD8+ infiltration in CRC. Next, by in vitro and in vivo, we found that increased APOL3 expression was positively correlated with sensitivity to ferroptosis and antitumor ability of CD8+ T cells. Next, we demonstrated that APOL3 can binds LDHA and promote its ubiquitylation-related degradation. Then, based on in vivo analysis and tumor specimen, we discovered the APOL3-LDHA axis can facilitate the tumor ferroptosis and cytotoxic ability of CD8+ T cells through increased IFNγ and decreased lactic acid concentration.

**Conclusion:** The present study demonstrated that APOL3 promotes ferroptosis and immunotherapy in colorectal cancer cells. The present work provides us with a novel target to overcome drug resistance to ferroptosis and immunotherapy.

## Introduction

Colorectal cancer (CRC) is common around the world 1, 2. Development of early detection modalities has improved the survival of patients with colorectal cancer and surgical resection is considered the only chance to cure CRC 3, 4. However, In China, CRC still ranks as the third leading cause of cancer-associated mortality and late-stage colorectal cancer responds poorly to most chemotherapeutic agents 5. Immune Checkpoint blockade therapy (ICB) is a new therapeutic approach to CRC. Based on the results of Keynote-177 trial, PD-1 blockade was approved to treat only a small subset of patients with CRC (microsatellite instability high or mismatch repair deficient) 6, 7. Unfortunately, approximately the other 85% of CRC with microsatellite stability (MSS) or mismatch repair proficient (pMMR) are still not responsive to ICB therapy. Hence, it is urgent to unveil the molecular mechanism of tumor resistance to ICB therapy and demonstrated novel treatment strategies.

CD8+ T cells play a central role in antitumor immunity and improvement of their functions is the key strategy to facilitate ICB therapy 8, 9. Targeting tumor cell death was regarded as an effective way to promote antitumor immunity through damage associated molecular patterns (DAMPs) 10, 11. Ferroptosis is a novel form of regulated cell death characterized by iron-dependent accumulation of lipid reactive oxygen species (ROS) to lethal levels 12. In recent years, accumulated progress has been made in the understanding of pathways on cancer sensitivity to ferroptosis 13-16. A biologically plausible hypothesis is that ferroptotic cells communicate with immune cells through a set of signals, such as the “find me” and “eat me” signals produced during cell death 17. Our previous evidence demonstrated that tumor ferroptosis status (consisting of GPX4, NOX1 and ACSL4) can reflect enhanced CD8+ T cell infiltration based on CRC specimen 18. The key molecular pathways of how ferroptosis enhance CD8+ T cell infiltration still remain obscure.

In this study, we firstly conducted a three-phase screen to identify apolipoprotein L3 (APOL3) as the potential modulator at the crossroad between ferroptosis and CD8+ T cell infiltration; then, by in vitro and in silicon analysis, we validated APOL3 as an important ferroptosis and immune-oncological modulator; next, through mRNA sequence, metabolomics analysis and combining protein-to-protein interaction (PPI) analysis, we identify APOL3 can negatively regulated LDHA at the protein level to facilitate ferroptosis and antitumor activity of CD8+ T cell; finally, by in vivo analysis, ferroptosis inducer RSL3 can enhance the anticancer effects of PDL1 inhibitor and overexpression of APOL3 was found to further improve the synergetic effect of RSL3 and PD-1 inhibitor in CRC.

## Methods and Materials

### Tumor samples and clinical characteristics

Validation cohort included 150 CRC microsatellite instability (MSS) patients with paired primary tumor and normal epithelium tissues. All samples were collected with institutional review board approval from Zhongshan Hospital, Fudan University. Inclusion criteria was as follows: a) age elder than 18; b) informed consent was obtained from each patient; c) no pre-operative adjuvant treatment, including targeted therapy, systematic chemotherapy, radiotherapy; d) received resection of colorectal cancer; e) pathological examination confirmed adenocarcinoma, and the immunohistochemistry analysis determined no loss of expression of any mismatch repair (MMR) protein (including MLH1, MSH2, MSH6, PMS1, PMS2). Exclusion criteria were as follows: Exclusion Criteria: a) History of other kind of cancers; b) diagnosed as Hereditary colorectal cancer or lynch syndrome. Informed written consent was obtained from all human participants. The ethics approval ID was B2017-166R.

### Transcriptome sequencing

Total RNA was isolated from frozen tumor tissues using a standard TRIzol protocol. Paired-end sequencing (2×100 bp) was carried out with a BGI-500 instrument (BGI) to obtain at least 20 million reads for each sample. The sequence data were processed and mapped to the human reference genome (hg19) using Bowtie2. Gene expression was quantified to fragments-per-kilobase per million mapped fragments (RPKM) using RNA-Seq by expectation-maximization (RSEM).

### Coimmunoprecipitation

Cells were lysed in IP buffer containing protease inhibitors (10 μg/mL leupeptin, 10 μg/mL aprotinin and 1 mM phenylmethylsulfonyl fluoride), mixed with 1 μg target protein antibody and 20 μL protein G-Sepharose (Thermo Fisher #101242), incubated overnight and eluted by boiling with SDS loading buffer. The eluted samples were detected by SDS-PAGE followed by Coomassie staining (Colloidal Blue Staining Kit, Invitrogen, #LC6025). For mass spectrometry, IP samples were eluted by shaking in 8 M urea and 100 mM Tris-Cl (pH 8.0) and then analyzed by mass spectrometry.

### Statistics

Statistical analyses were performed using the SPSS statistical package (22.0; SPSS), R studio (R Project for Statistical Computing) and Prism 6 (GraphPad Prism). The correlations between continuous variables were analyzed using the Spearman rank correlation test and the chi-squared test. Overall survival (OS) analyses were carried out using the Kaplan-Meier method, and the results were compared using a log-rank test. A multivariable Cox proportional hazards model predicting OS was performed using backward stepwise selection. Risk factors were expressed as the hazard ratio [HR, 95% confidence interval (CI)]. Statistical significance was defined as a P-value less than 0.05.

## Results

### Integrated screen identifies APOL3 as the significant modulator between ferroptosis and immune-activation in Colorectal cancer

**Figure 1:**
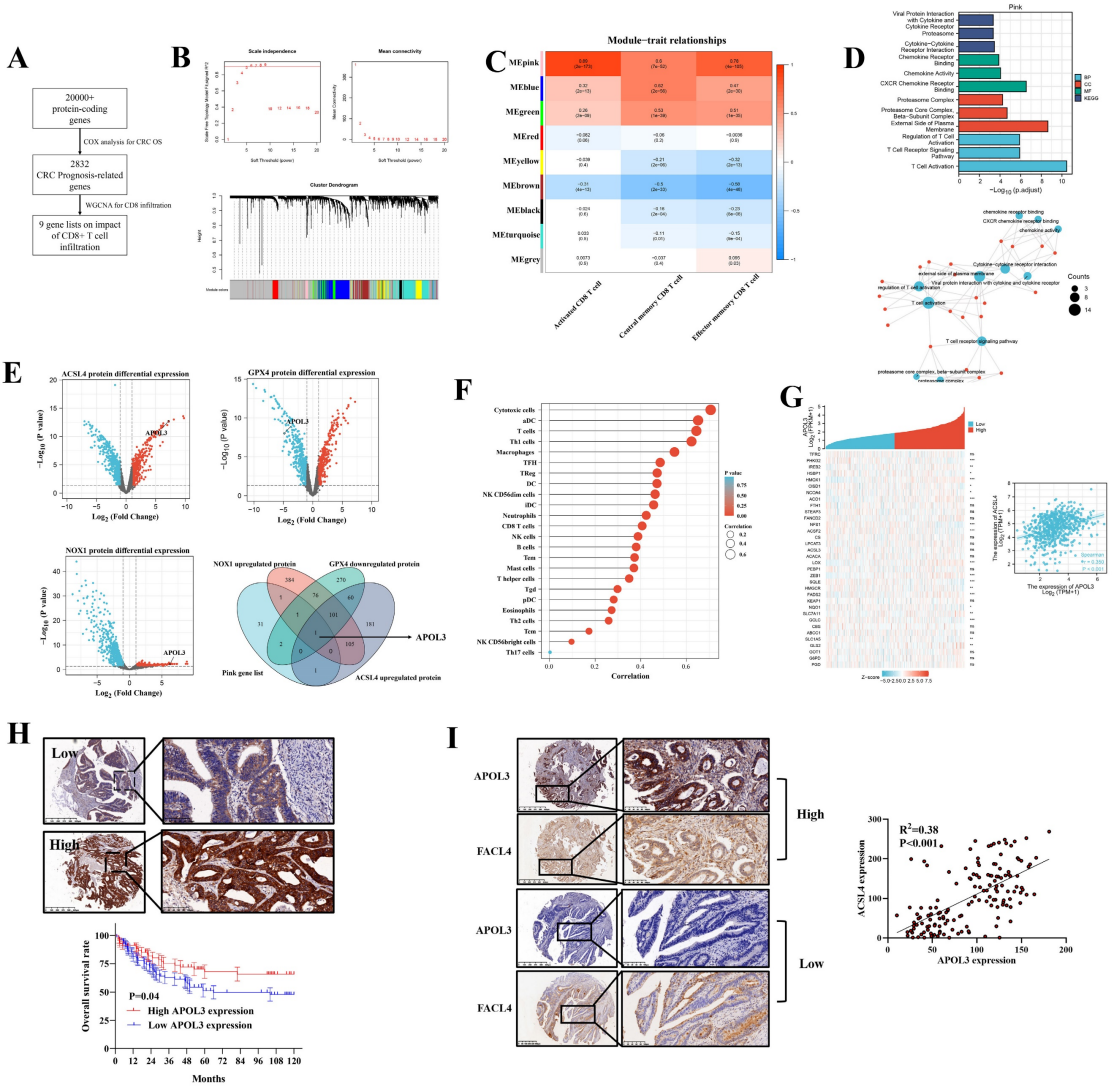
integrated screen identifies APOL3 as significant modulator for ferroptosis and antitumor immunity in CRC. **(A)** to **(C)**: to identify novel ferroptosis and antitumor immunity modulators in CRC, we performed a three-stage screen analysis** (A)** first, we performed COX analysis to screen prognosis-related genes in CRC patients, 2832 genes were screened; **(B)** second, we performed WGCNA analysis to demonstrate 9 modules correlated with tumor related CD8+ T cells infiltration; **(C)** 9 modules were separately blanked with different color marks; **(D)** validation of the Pink gene list on tumor immunity by GO analysis; **(E)** third, differential protein expression analysis from CPTAC database, based on overlapping gene list, APOl3 was selected; **(F)** SsGSEA analysis demonstrated the immune-activation role of APOL3 in CRC samples; **(G)** mRNA expression relation between APOL3 and ferroptosis-related markers was shown, a linear regression relationship was demonstrated between APOL3 and ACSL4 (P<0.05). **(H)** IHC assay was performed on TMA from Zhongshan Hospital colorectal cancer center and representative images were shown, low expression of APOL3 indicates a poor prognosis in CRC patients from Zhongshan Hospital cohort; **(I)** IHC assay demonstrated the positive correlation between APOL3 and FACL4. **Abbreviations:** CRC, colorectal cancer; WGCNA, weighted gene co-expression network analysis; IHC, immunohistochemistry; CPTAC, Clinical Proteomic Tumor Analysis Consortium; TMA, tissue microarray; SsGSEA, single sample gene set enrichment analysis.

Our previous analysis demonstrated that ferroptosis (combining of GPX4, NOX1 and ACSL4) indicated tumor immune-activation in CRC 18. Thus, we performed a three-phase screen to identify the potential molecule targets. First, based on TCGA data, we conducted COX analysis to demonstrate 2832 genes significantly related to CRC prognosis (**Figure 1A** and **dataset S1 and S2**). Second, we identified 9 modules of WGCNA related to CD8+ T cell infiltration rate (**Figure 1B**) and carried out module-trait relationship analysis (**Figure 1C**). Although multiple modules were detected to be related to CD8+ T cell infiltration at varying degrees, we found that the MEpink module confers to a significant correlation and the highest absolute value of r (r = 0.89) (**Figure 1C**) and the MEpink module was further confirmed by Gene ontology (GO) analysis (**Figure 1D** and **Figure S1**). Third, based on another independent Clinical Proteomic Tumor Analysis Consortium (CPTAC) proteomics CRC data, differential expression analysis (DEA) was performed to determine relative molecules for GPX4 (**dataset S3**), NOX1 (**dataset S4**) and ACSL4 (**dataset S5**), overlapping the statistically significant genes and MEpink modules, APOL3 was screened to be potentially key gene modulating ferroptosis and enhanced tumor infiltrating CD8+ T cells' activity (**Figure 1E**) and the mRNA differential analysis was shown in **Figure S2**.

### APOL3 was functionally and clinically important for colorectal cancer

The correlation between APOL3 expression and immune cells infiltration was shown in **Figure 1F** and **Figure S3**. We further retrieved MCPCOUNTER 19, CIBERSORT 20, Xcell 21 and Quantiseq 22 to define CD8+ T cell significance of APOL3 in CRC (**Figure S4**). Besides, the ferroptotic role of APOL3 in CRC was also evaluated in **Figure 1G,** whereas the rest of APOL family proteins was found with less correlation with most of the known ferroptotic markers (**Table S1**) in **Figure S5** (APOL5 was excluded for the low expression in CRC tissues).

The clinical relevance of APOL3 in CRC was also assessed from TCGA data, mRNA expression of APOL3 down-regulated in CRC tissues (P<0.001) and low expression of APOL3 statistically correlates with more lymph node metastasis and distant metastasis (**Figure S6**). Moreover, we performed immunohistochemistry (IHC) staining with validated antibodies against APOL3 (**Figure 1H**) on a CRC tissue microarray from Zhongshan Hospital, Fudan University. Our analysis showed that low expression of APOL3 was associated with a poor prognosis of patients with CRC (**Figure 1H**). The correlation between the expression of APOL3 and clinical characteristics is shown in **Table S2**. Low expression of APOL3 was significantly correlated with higher T stage (P=0.001, **Table S2**). More importantly, Expression of APOL3 was positively correlated with expression of FACL4 (P<0.001, R^2^=0.38, **Figure 1I**). Taken together, these findings suggest a clinically significant role of APOL3 in CRC.

### Overexpression of APOL3 inhibits proliferation and enhances RSL3-induced ferroptosis in colorectal cancer

**Figure 2:**
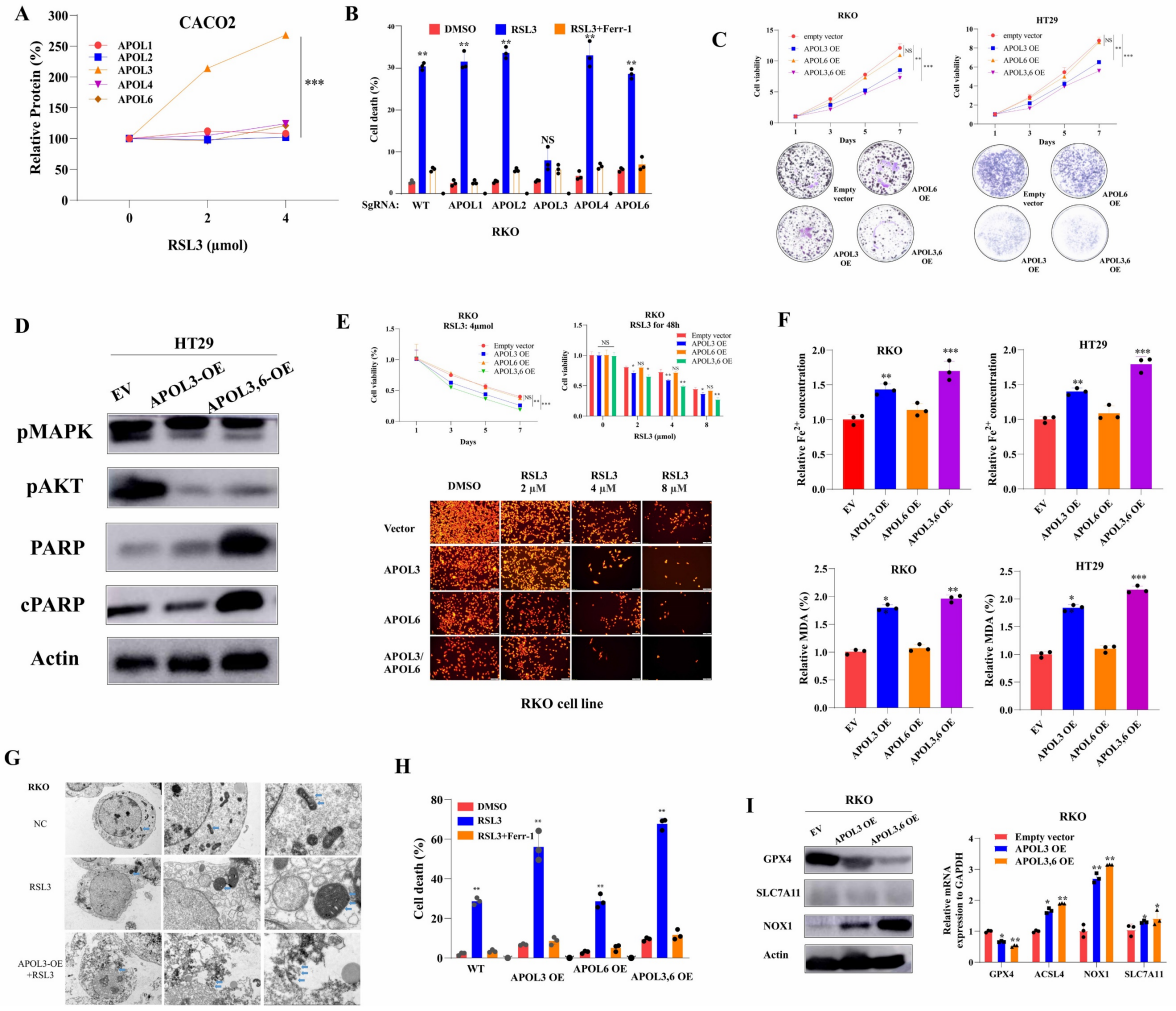
Overexpression of APOL3 inhibits cell proliferation and promotes ferroptosis in CRC cells. **(A)** expression of APOL3 was up-regulated after treatment of RSL3, while the level of APOL1, APOL2, APOL4 and APOL6 was slightly changed; **(B)** then we transfected RKO colon cancer cells with APOL1-KO, APOL2-KO, APOL3-KO, APOL4-KO and APOL6-KO, among APOL family, only APOL3-KO RKO cell death rate was significantly reduced; **(C)** compared to normal controls by CTG and clone formation assay, overexpression of APOL3 significantly inhibits cell proliferations in RKO and HT29 cells; **(D)** Western Blots demonstrated APOL3 regulated CRC cell proliferation and death; **(E)** by CTG and TMRE assay, RKO^APOL3-OE^ cells were more sensitive to RSL3 upon exposure to increasing RSL3 concentrations; **(F)** iron accumulation and lipid ROS production was significantly up-regulated with overexpression of APOL3 in CRC cells; **(G)** electron microscope images demonstrated significant cellular changes in RKO; **(H)** inducing ferroptosis with RSL3, the cell death rate between controls and APOL3-OE was significantly increased; **(I)** Next, we performed western blotting and qRT-PCR to evaluate both protein and mRNA levels of GPX4, NOX1 and ACSL4 and the significant changes were observed. **Abbreviation:** CRC, colorectal cancer; CTG, CellTiter-Glo luminescent assay; TMRE, Tetramethylrhodamine Ethyl Ester assay; qRT-PCR, qualitative reverse transcriptase polymerase chain reactions.

Based on above bioinformatics and clinical analysis, we tested whether ferroptosis inducer RSL-3 can affect the expression of APOL3. As was shown in **Figure 2A**, only expression of APOL3 among APOL family was sharply up-regulated after treatment of RSL3 and the level of APOL1, APOL2, APOL4 and APOL6 was slightly changed. Moreover, we transfected RKO colon cancer cells with APOL1-KO, APOL2-KO, APOL3-KO, APOL4-KO and APOL6-KO. Upon RSL3 treatment, comparing to normal control cells, only APOL3-KO RKO cell death rate was significantly reduced, while the other 4 transfected cells demonstrated no differences on cell death rate (**Figure 2B**).

To investigate the role of APOL3 in CRC cell lines, we generated APOL3-KO HCT-116 and RKO cells using SgRNA (**Figure S7**) and constructed APOL3-OE HT29 and CACO2 cells (**Figure S8**). For CRC cell line HT29 and CACO2, overexpression of APOL3 inhibits cell growth, as measured by cell viability and colony formation assays in **Figure 2C**. Accordingly, Western Blots demonstrated that the expression of proteins reflecting proliferation and death also changed (**Figure 2D**). As APOL3 expression was significantly correlated with ferroptosis markers, we further evaluated ferroptotic role of APOL3 in a CRC cell line. As shown in **Figure 2E**, RKO^APOL3-OE^ cells were more sensitive to RSL3 upon exposure to increasing drug concentrations. Furthermore, overexpression of APOL3 significantly influenced RSL3-induced ferroptotic events, including iron accumulation and lipid ROS production (**Figure 2F**), then Transmission Electron Microscope (TEM) demonstrated significant cellular changes in RKO (**Figure 2G, marked in blue arrow**). After inducing ferroptosis with RSL3, the cell death rate between controls and APOL3-OE was significantly increased (**Figure 2H**); As a negative control, overexpression of APOL6 demonstrated slight changes on cell proliferation or ferroptosis in CRC cell lines (**Figure 2C-2G**). Next, we performed western blotting and qRT-PCR to evaluate both protein and mRNA levels of GPX4, NOX1 and ACSL4 after transfection of APOL3-OE, the significant changes were observed (**Figure 2I**). Together, these results indicate that APOL3 is essential for the CRC cell line progressive phenotype and overexpression of APOL3 promotes ferroptosis in CRC cell lines.

### Knock-out of APOL3 inhibits RSL3-induced ferroptosis in colorectal cancer

**Figure 3:**
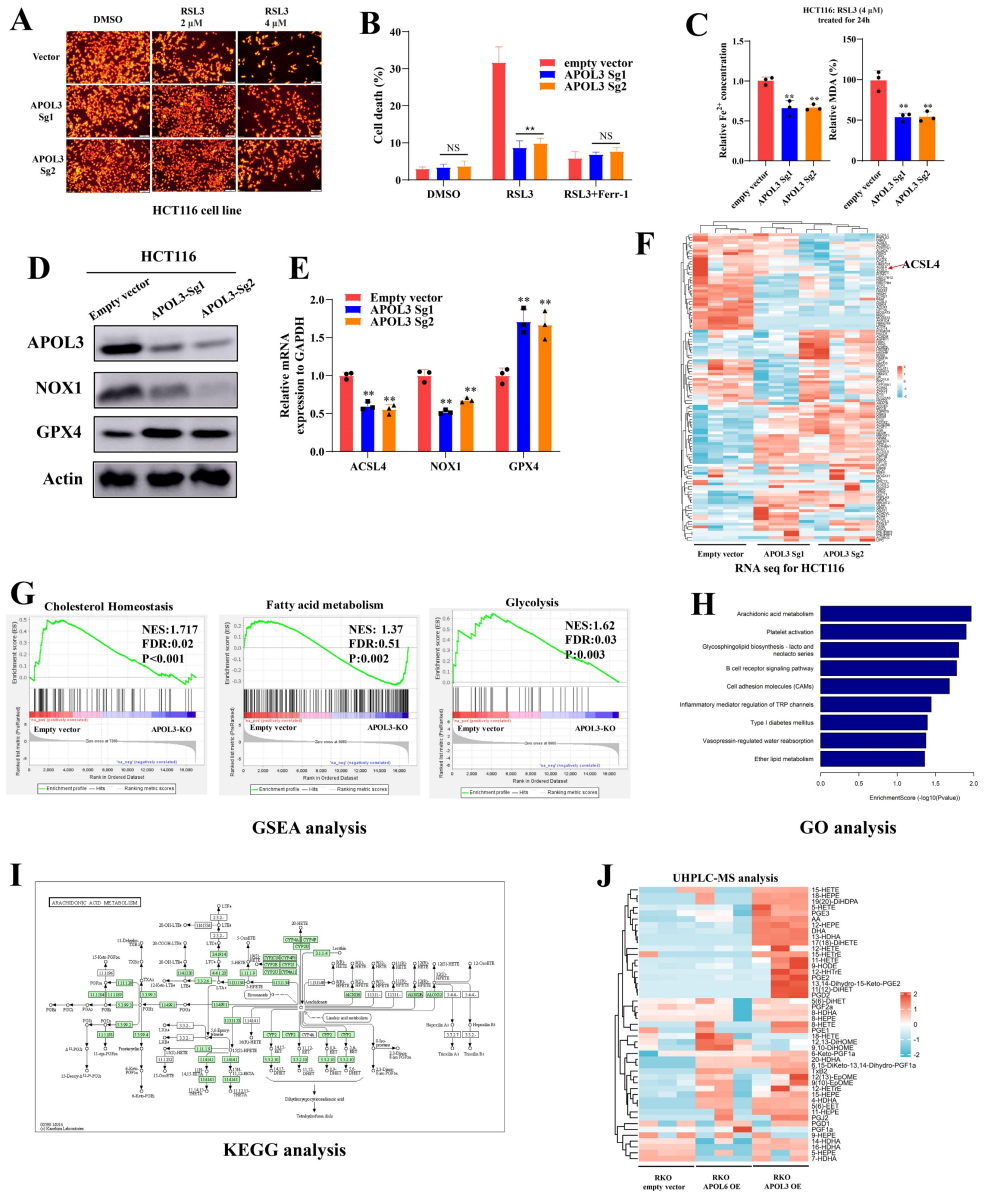
Knock-out of APOL3 inhibits RSL3-induced ferroptosis and regulates lipid metabolism in CRC. **(A)** we constructed APOL3-Knock out in HCT116 cell line, TMRE analysis demonstrated that knock-out of APOL3 significantly inhibits RSL3-induced ferroptosis; **(B)** cell death rate of APOL3-KO cells was markedly reduced with the treatment of RSL3; **(C)** the volume of Fe^2+^ and the MDA rate was also significantly decreased; western blotting (**D**) and qRT-PCR (**E**) results confirmed that HCT116^APOL3-KO^ inhibits RSL3-induced ferroptosis in CRC; **(F)** transcriptome sequencing of controls and APOL3-KO HCT116 cells was performed and the mRNA expression signature of lipid metabolism genes was shown; **(G)** GSEA of APOL3-Sg1 and APOL3-Sg2 involved the gene sets of “Cholesterol Homeostasis”, “Fatty acid metabolism” and “Glycolysis”; **(H)** and **(I)** GO and KEGG analysis revealed that APOL3 loss significantly enriched the expression of genes linked to Arachidonic acid metabolism; **(J)** MS analysis of majority oxidized fatty acids were significantly upregulated in APOL3-OE CRC cells. **Abbreviations:** CRC, colorectal cancer; CTG, Cell Titer-Glo luminescent assay; TMRE, Tetramethylrhodamine Ethyl Ester assay; GO, Gene Ontology; GSEA, gene set enrichment analysis; OE, overexpression; KO, knockout.

Next, we constructed APOL3-Knock out in HCT116 cell line, Tetramethylrhodamine Ethyl Ester (TMRE) analysis demonstrated that knock-out of APOL3 significantly inhibits RSL3-induced ferroptosis (**Figure 3A**). Indirect data also revealed that cell death rate was significantly reduced after knock out of APOL3 (**Figure 3B**). Moreover, RSL3-induced ferroptosis-related events such as the volume of Fe^2+^ and the MDA rate was also significantly decreased (**Figure 3C**). Finally, western blotting (**Figure 3D**) and qRT-PCR (**Figure 3E**) results confirmed that HCT116^APOL3-KO^ inhibits RSL3-induced ferroptosis in CRC.

### APOL3 functions as a regulator for lipid metabolism in colorectal cancer cells

To better understand APOL3-mediated signal circuits, we performed transcriptome sequencing of controls and APOL3-KO HCT116 cells. The mRNA expression signature is shown in **Figure 3F** (**Dataset S6**). In addition, Gene set enrichment analysis (GSEA) of APOL3-Sg1 and APOL3-Sg2 significantly involved the gene sets of “Cholesterol Homeostasis” (NES:1.717, FDR:0.02, P<0.001), “Fatty acid metabolism” (NSE: 1.37, FDR: 0.51, P:0.002) and “Glycolysis” (NES: 1.62, FDR:0.03, P:0.003). Next, we performed Gene ontology (GO) analysis and observed that APOL3 loss significantly enriched the expression of genes linked to Arachidonic acid metabolism (**Figure 3H** and **Figure 3I**). Moreover, Mass spectrometry (MS) data analysis of oxidized fatty acids also were detected and quantified from the cell samples (**Figure 3J**). The quantitative results were showed in **Dataset S7** and the Extracted Ion Chromatograms (EICs) for controls and APOL3-OE were demonstrated in **dataset S8**.

### Transcriptome sequencing and Protein-protein interaction network prediction revealed a potential downstream substrate of APOL3 in colorectal cancer cells

**Figure 4:**
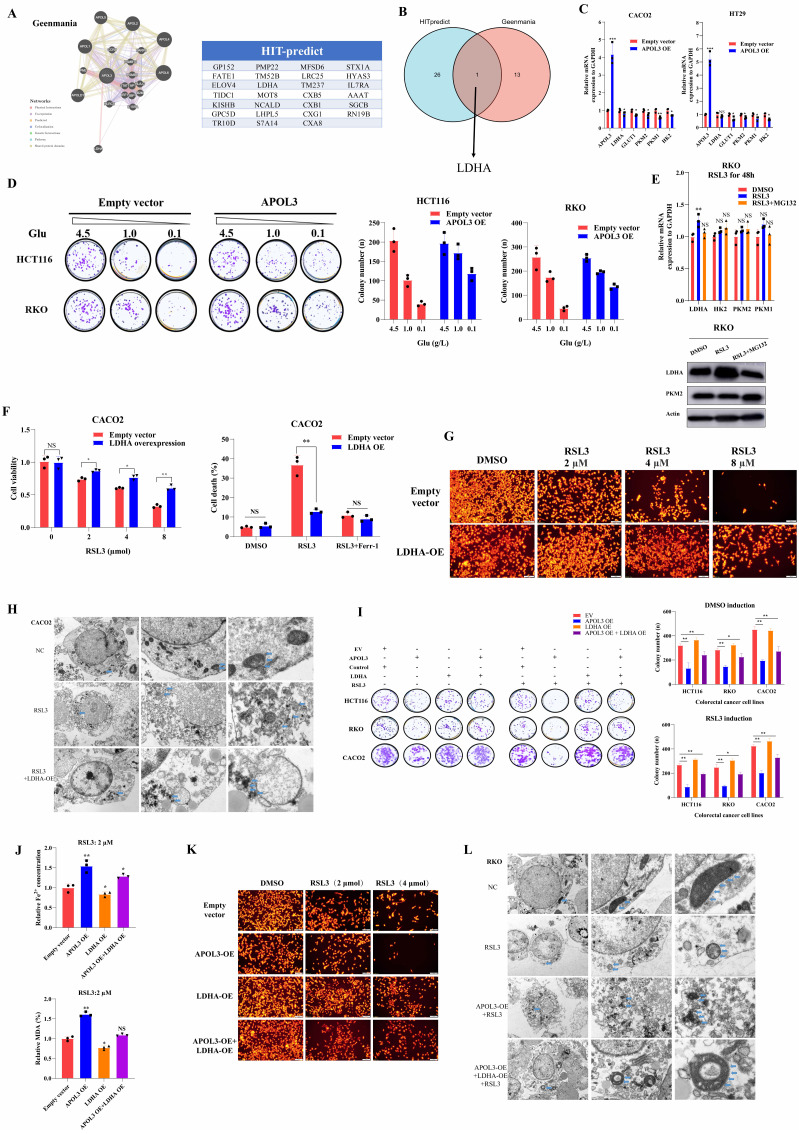
APOL3-LDHA axis regulated ferroptosis in CRC. **(A)** PPI prediction from Gennmania and HIT-predict was performed **(B)** LDHA was screened as the only overlapped gene; **(C)** mRNA levels changes were shown after alteration of APOL3; **(D)** biological effect of APOL3 were highly addicted to glucose, and gradient deprivation of glucose inhibited their viability compared to negative controls; **(E)** treatment of RSL3 upregulated mRNA the expression of LDHA and this effect can be partly diminished with addition of MG132; **(F)** by CTG assay and cell death rates evaluation, overexpression of LDHA inhibits RSL3-induced ferroptosis in CACO2; **(G)** TMRE analysis demonstrated that overexpression of LDHA significantly inhibits ferroptosis of colon cancer cells; **(H)** by electron image microscopy, LDHA-OE significantly prevent cellular responses from RSL3 in CACO2 cells; **(I)** by clone formation analysis of CRC cell lines HCT116, RKO and CACO2, overexpression of LDHA can rescue APOL3-OE induced inhibition of cell proliferation and cell death; **(J)** concentration of Fe^2+^ and MDA (%) was also rescued after LDHA was overexpressed in CRC cells; **(K)** TMRE analysis found that LDHA-OE can rescue APOL3-OE-induced sensitivity to ferroptosis; **(L)** similar cellular response was shown by electron images. **Abbreviations:** CRC, colorectal cancer; PPI, protein-protein interaction; CTG, Cell Titer-Glo luminescent assay; TMRE, Tetramethylrhodamine Ethyl Ester assay.

Given the metabolic functions validated by MS analysis and transcriptome sequence after alteration of APOL3 in CRC cell lines, we further wonder what the downstream pathway APOL3 regulated is for the ferroptosis phenotype. Thus, we doubly performed in silicon protein-protein interaction (PPI) network analysis to predict the potential substrate; based on Geenmania 23 and HIT-prediction 24 analysis,** dataset S9** and** Figure 4A** concluded the interacting protein and only the L-lactate dehydrogenase A (LDHA) was overlapped. The protein levels of LDHA were negatively regulated by APOL3 in three CRC cell lines (CACO2, HT29 and HCT116) in **Figure 4B**, while the mRNA levels were not significantly changed after the alteration of APOL3 (**Figure 4C**). Combining the results of the transcriptome sequencing, MS analysis, in silicon PPI prediction and preliminary verification, we considered the LDHA to be a potential downstream substrate of APOL3 in CRC cell line and the regulated process may be at post-translation level.

### APOL3 inhibits cell proliferation and ferroptosis via negatively regulating LDHA

Compared to negative controls, we constructed APOL3-overexpressed CRC cells, next, by colony formation assays, With the decrease of glucose sugar concentration in the culture medium, we demonstrated that viability of APOL3-OE cells was more tolerance to the gradient deprivation of glucose (**Figure 4D**). To validate the ferroptotic role of LDHA, we tested the mRNA and protein level of LDHA and its family genes with treatment of RSL3 in CRC cell line. As was shown in **Figure 4E**, induction of RSL3 can significantly upregulate the expression of LDHA and, interestingly, this effect can be partly diminished with addition of MG132 (the inhibitor of Proteasome system). Overexpression of LDHA inhibits RSL3-induced ferroptosis through evaluating cell viability and cell death rates on colon cancer cell line CACO2 (**Figure 4F**). Moreover, TMRE analysis (**Figure 4G**) and TEM (**Figure 4H**) analysis demonstrated that overexpression of LDHA significantly inhibits ferroptosis of colon cancer cells. Next, by colony formation analysis of CRC cell lines HCT116, RKO and CACO2, overexpression of LDHA can rescue APOL3-OE induced inhibition of cell proliferation and ferroptosis (**Figure 4I, Figure S9 and Figure S10**). Accordingly, concentration of Fe^2+^ and MDA (%) was also rescued after LDHA was overexpressed in colon cancer cell lines (**Figure 4J**). Furthermore, TMRE analysis (**Figure 4K**) and TEM analysis (**Figure 4L**) demonstrated similar results. Taken together, these phenotypic results proved that, in CRC cell lines, the APOL3 promotes ferroptosis and inhibits cell proliferation via LDHA.

### APOL3 induces proteasome-dependent ubiquitination of LDHA

**Figure 5:**
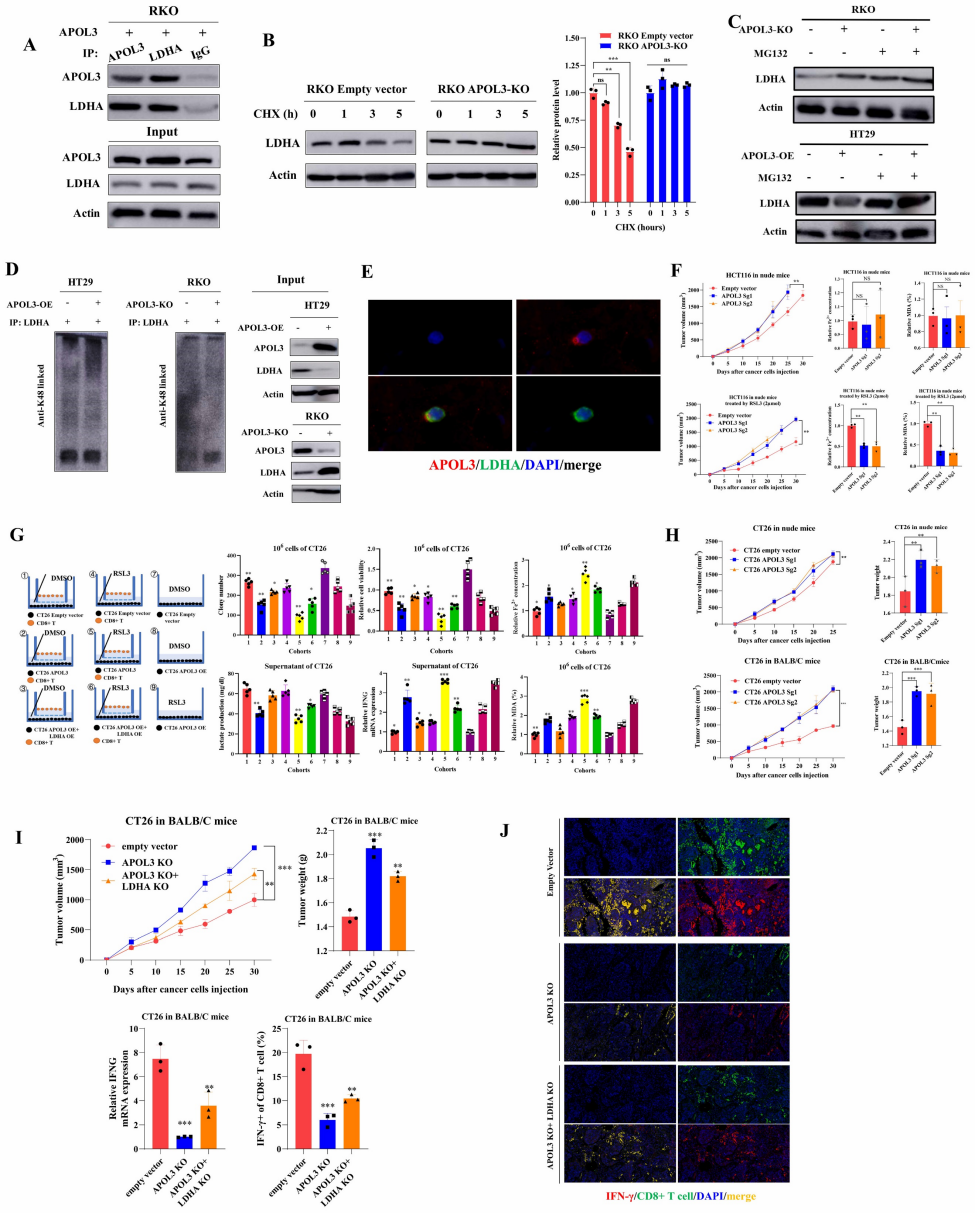
APOL3 promotes the ubiquitylation-related degradation of LDHA and the antitumor ability of CD8+ T cells in CRC in vitro and in vivo. **(A)** Reciprocal IP-western analysis revealed that APOL3 directly interacted with LDHA; **(B)** we pretreated cells with CHX to determine the stability of LDHA and results showed that the half-life periods of LDHA were much longer in CRC cells with APOL3 knock-out compared to control cells; **(C)** Western blotting analysis found that LDHA was significantly increased in APOL3-knockout RKO cells, whereas suppressed LDHA levels were seen in cells with APOL3 overexpression, and this effect was diminished by treatment of MG132; **(D)** Co-IP using total K48-linked poly-ubiquitination antibody identified that overexpression of APOL3 promoted K48-linked ubiquitination of LDHA in HT29 and RKO cells; **(E)** IF analysis found APOL3 signal significantly overlapped with LDHA on cytoplasm in CRC cells; **(F)** In vivo analysis with subcutaneous xenograft models were constructed, APOL3-knockout of HCT116 cells promoted tumor growth when compared to their negative controls **(left top)**; concentration of Fe^2+^ and MDA (%) in nude mice were not significantly altered (**right top**); with the treatment of ferroptosis inducer RSL3, APOL3 knockout significantly led to significant increase of tumor volume in vivo **(left down)**; knock-out of APOL3 significantly inhibits the expression of Fe^2+^ and percent of MDA (**right down**); **(G)** In vitro, mouse-derived CT26 colon cells were indirectly co-cultured with CD8+ T cells with different treatment groups, overexpression of APOL3 markedly increased the efficacy of CD8+ T cell with higher lactate concentration and IFNG expression and can be diminished after co-overexpression of APOL3 and LDHA; **(H)** subcutaneous xenograft models of CT26 were created and knock-out of APOL3 promotes CT26 growth in both nude mice and BALB/c mice; the tumor volume difference was larger in BALB/c immune-competent mice; **(I)** APOL3-LDHA axis effect on BALB/c mice is shown, knockout of LDHA partly diminished tumor-promoting effect of APOL3-KO in CT26 cells with slight increase of IFNG mRNA expression and IFNγ+ CD8+ T cells; **(J)** IF analysis revealed similar results. **Abbreviations:** CRC, colorectal cancer; CHX, cycloheximide; IP, immunoprecipitation; IF, immunofluorescence; Co-IP, co-immunoprecipitation.

In light of previous findings, to identify the molecular mechanistic basis by which APOL3 promotes ferroptosis through LDHA, we next performed reciprocal IP-western analysis to identify APOL3-LDHA interactions. As was shown in Figure 5A, APOL3 could directly interact with LDHA. As we know, ubiquitination is instrumental in the regulation of protein expression. Thus, we firstly pretreated cells with cycloheximide (CHX) to determine the stability of LDHA. Results showed that the half-life periods of LDHA were much longer in CRC cells with APOL3 knock-out than were in control cells (**Figure 5B**). We then evaluated the protein levels of LDHA and found that LDHA was significantly increased in APOL3-knockout RKO cells, whereas suppressed LDHA levels were seen in cells with APOL3 overexpression, next, we treated cells with MG132, results revealed that MG132 treatment could significantly increase the LDHA protein expression in control cells, while only a slight upregulation of LDHA was observed in cells with APOL3 knockout (**Figure 5C**). These results suggested that APOL3 might modulate the stability of LDHA in a proteasome-dependent manner. In support of the endogenous ubiquitination of LDHA, Co-IP using total K48-linked poly-ubiquitination antibody was then incubated with RKO and HT29 cell lysates, we identified that overexpression of APOL3 could increase K48-linked poly-ubiquitination of LDHA and the interaction between LDHA and APOL3 in both cells (**Figure 5D and Figure S11**). Furthermore, we analyzed the subcellular location of APOL3 and LDHA by Immunofluorescence (IF) analysis found APOL3 signal significantly overlapped with LDHA on cytoplasm in CRC cells (**Figure 5E**). Taken together, these results indicated that APOL3-LDHA interaction could induce LDHA proteasome-dependent ubiquitination and degradation in CRC cells.

### APOL3 inhibits growth and promotes ferroptosis of colorectal cancer cells in vivo

In light of our in vitro findings, we tested the functions of APOL3 in proliferation in vivo with subcutaneous xenograft models at first. Results showed HCT116 APOL3-knockout cells promoted tumor growth by 42% when compared to their negative controls (**Figure 5F left top**). Besides, knock-out of APOL3 did not influence the ferroptotic events such as concentration of Fe^2+^ and MDA (%) in nude mice (**Figure 5F right top**). Furthermore, with the treatment of ferroptosis inducer RSL3, APOL3 knockout significantly led to significant upregulation of tumor volume (**Figure 5F left down**). Accordingly, knock-out of APOL3 significantly inhibits the expression of Fe^2+^ and percent of MDA (**Figure 5F right down**). Moreover, we chose to perform the subcutaneous xenograft models of APOL3-overexpressed HT29 CRC cells, consistent with previous findings, overexpression of APOL3 significantly inhibits tumor volume (**Figure S12**) and promotes the efficacy of RSL3 (**Figure S13**); APOL3-overexpressed HT29 of Fe^2+^ concentration (**Figure S14**) and percent of MDA (**Figure S15**) was markedly increased by treatment of RSL3 in vivo. Collectively, these results supported the view that APOL3 played anti-carcinogenic and ferroptosis-promoting roles in CRC cells in vivo.

### Co-culture and in vivo analysis demonstrate immune-activated effect of APOL3-overexpression in colorectal cancer cell lines

Previous report demonstrated that LDHA blunts tumor immunosurveillance by CD8+ T cells^25^. In light of our above findings of APOL3-LDHA axis, we performed both in vitro and in vivo analysis to demonstrate the effect of APOL3-overexpressed CRC cells on the anti-tumor ability of CD8+ T cell. In vitro, mouse-derived CT26 colon cells were indirectly co-cultured with CD8+ T cells with different treatment groups. As was shown in **Figure 5G (both left parts and right parts)**, in DMSO treatment cohorts, APOL3 overexpression markedly increased the efficacy of CD8+ T cell with higher lactate concentration and IFNG expression (**cohort 1** and **cohort 2**) and can be diminished after co-overexpression of APOL3 and LDHA (**cohort 3**); on the other hand, in RSL3 treatment cohorts (**Figure 5G right parts**), we firstly find in vitro that treatment of RSL3 can significantly enhance antitumor effect of CD8+ T cell with higher IFNG mRNA expression (**cohort 4** and **cohort 5**). Besides, Fe^2+^ concentration and percent of MDA also changed accordingly (**Figure 5G**).

Next, in vivo, we created subcutaneous xenograft models of CT26 and consistent with previous findings, knock out of APOL3 promotes CT26 growth in both nude mice and BALB/c mice (**Figure 5H**), however, with same number of CT26 cells injected, the tumor volume difference was larger in BALB/c mice with normal immune system. We further APOL3-LDHA axis effect on BALB/c mice, as is shown in **Figure 5I**, knockout of LDHA partly diminished tumor-promoting effect of APOL3-KO in CT26 cells with slight increase of IFNG mRNA expression and IFNγ+ CD8+ T cells. IF analysis also revealed similar results in **Figure 5J**. Together, these data demonstrated that alteration of APOL3 of CT26 played influence on tumor volume through tumor microenvironment modulation.

### APOL3-LDHA axis enhances antitumor effect of PD1 blockade in vivo

**Figure 6:**
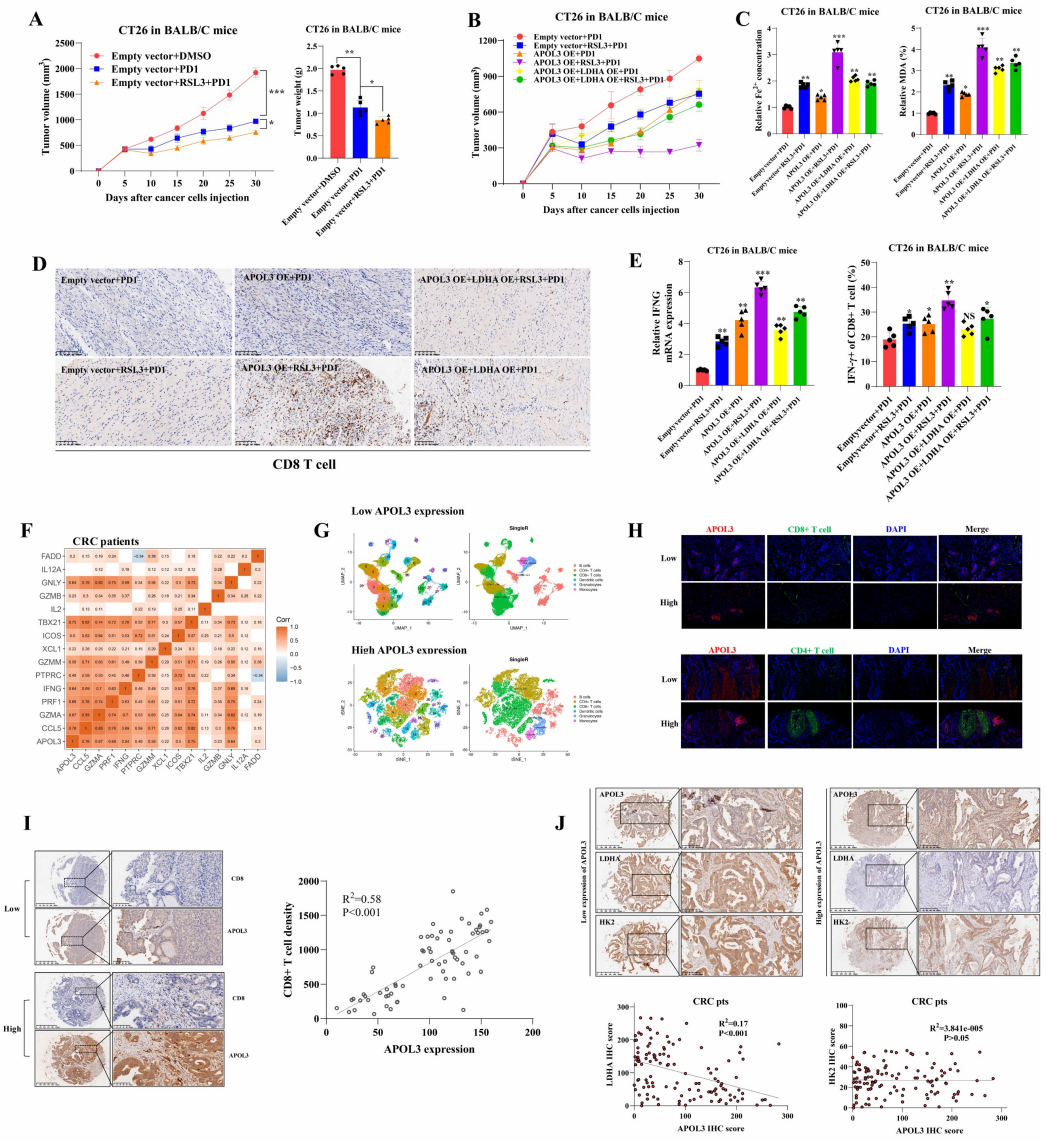
APOL3-LDHA axis enhances antitumor effect of PD1 blockade in vivo and APOL3 expression correlated with LDHA, CD8+ T cell infiltration and ferroptosis markers in CRC. **(A)** Xenograft models were divided into three different groups: DMSO cohort, PD1 cohort and RSL3+PD1 cohort and results showed although CT26 tumors with treatment of RSL3+PD1 were slightly smaller compared to their control mice; **(B)** APOL3-OE of CT26 with treatment of RSL3 significantly increased the antitumor ability of PD-1 inhibitor in vivo and this effect can be diminished by co-overexpression of LDHA; **(C)** Fe^2+^ expression and percent of MDA analysis were accordingly changed in different cohorts relative to **(B)**; **(D)** CD8+ T cells infiltration was also evaluated by IHC analysis and APOL3-OE plus RSL3 cohort led to upregulation of CD8+ T cell infiltrations; **(E)** APOL3-OE+RSL3+PD1 cohort tumor showed the largest IFNG expression and percent of IFNγ of CD8+ T cells compared to another 5 cohorts; **(F)** based on TCGA database, expression of APOL3 was positively associated with expression of many cytotoxic cytokine-related genes; **(G)** immune-infiltration reads by performing single-cell RNA sequence with 2 different CRC specimen (low and high APOL3 expression) was evaluated and high expression of APOL3 was positively correlated with antitumor-microenvironment; **(H)** IF analysis and IHC analysis **(I)** determined the positive correlation between APOL3 and CD8+ T infiltration and negative correlation between APOL3 and LDHA expression **(J)**. **Abbreviations:** CRC, colorectal cancer; DMSO, Dimethyl sulfoxide; TCGA, The Cancer Genome Atlas; IF, immunofluorescence; IHC, immunohistochemistry; TMA, tissue microarray.

In light of previous findings, we next created the subcutaneous xenograft models of CT26 cells in BALB/c mice. Xenograft models were divided into three different groups: DMSO cohort, PD1 cohort and RSL3+PD1 cohort. In **Figure 6A**, results showed although CT26 tumors with treatment of RSL3+PD1 were smaller compared to their control mice, RSL3 can only enhances the antitumor effect of PD1 blockade by 14%. Next, six different cohorts were created in CT26 Xenograft mice: Empty vector+PD1, Empty vector+RSL3+PD1, APOL3-OE+PD1, APOL3-OE+RSL3+PD1, APOL3-OE+LDHA-OE+PD1 and APOL3-OE+LDHA-OE+RSL3+PD1. Consistent with above findings, tumor volume analysis indicated that APOL3-OE of CT26 with treatment of RSL3 significantly increased the antitumor ability of PD-1 inhibitor in vivo and this effect can be diminished by co-overexpression of LDHA (**Figure 6B**). Accordingly, Fe^2+^ expression and percent of MDA analysis demonstrated similar results (**Figure 6C**). Furthermore, CD8+ T cells infiltration was also evaluated by IHC analysis and APOL3-OE plus RSL3 cohort led to upregulation of CD8+ T cell infiltrations (**Figure 6D**). Then, we chose to perform qRT-PCR analysis and Flow Cytometry to evaluate antitumor ability of CD8+ T cells in CT26 tumors. Not surprisingly, APOL3-OE+RSL3+PD1 cohort tumor showed the largest IFNG expression and percent of IFNγ of CD8+ T cells compared to another 5 cohorts (**Figure 6E**). Collectively, these results supported the view that APOL3-LDHA axis enhanced antitumor ability of CD8+ T cell-based treatment in CRC cells in vivo.

### Clinically, APOL3 expression correlated with LDHA, tumor microenvironment and ferroptosis markers in colorectal cancer

To verify why low APOL3 expression is associated with dampened antitumor function and poor prognosis, we analyzed the relationship between APOL3 and cytotoxic cytokine-associated gene expression in tumor-infiltrating CD8+ T cells in CRC patients from TCGA database. Results showed that the expression of APOL3 was positively associated with expression of many cytotoxic cytokine-related genes and with their transcription factors, such as IFNG, CCL5, GZMB, and TBX21 (**Figure 6F** and** dataset S10**). Next, we validated the immune-infiltration reads by performing single-cell RNA sequence with 2 different CRC specimen (low and high APOL3 expression) and found that high expression of APOL3 was positively correlated with antitumor-microenvironment (**Figure 6G**), another external validation was also performed in **Figure S16**. Furthermore, IF analysis of CRC specimen showed similar results and the negative correlation between APOL3 and LDHA expression (**Figure 6H**). Finally, IHC analysis based on CRC specimen revealed that APOL3 expression was positively correlated with CD8+ T cell infiltration with R^2^ of 0.58 (**Figure 6I**) and negatively correlated with tumor LDHA expression with R^2^ of 0.17 (**Figure 6J**).

**Figure 7:**
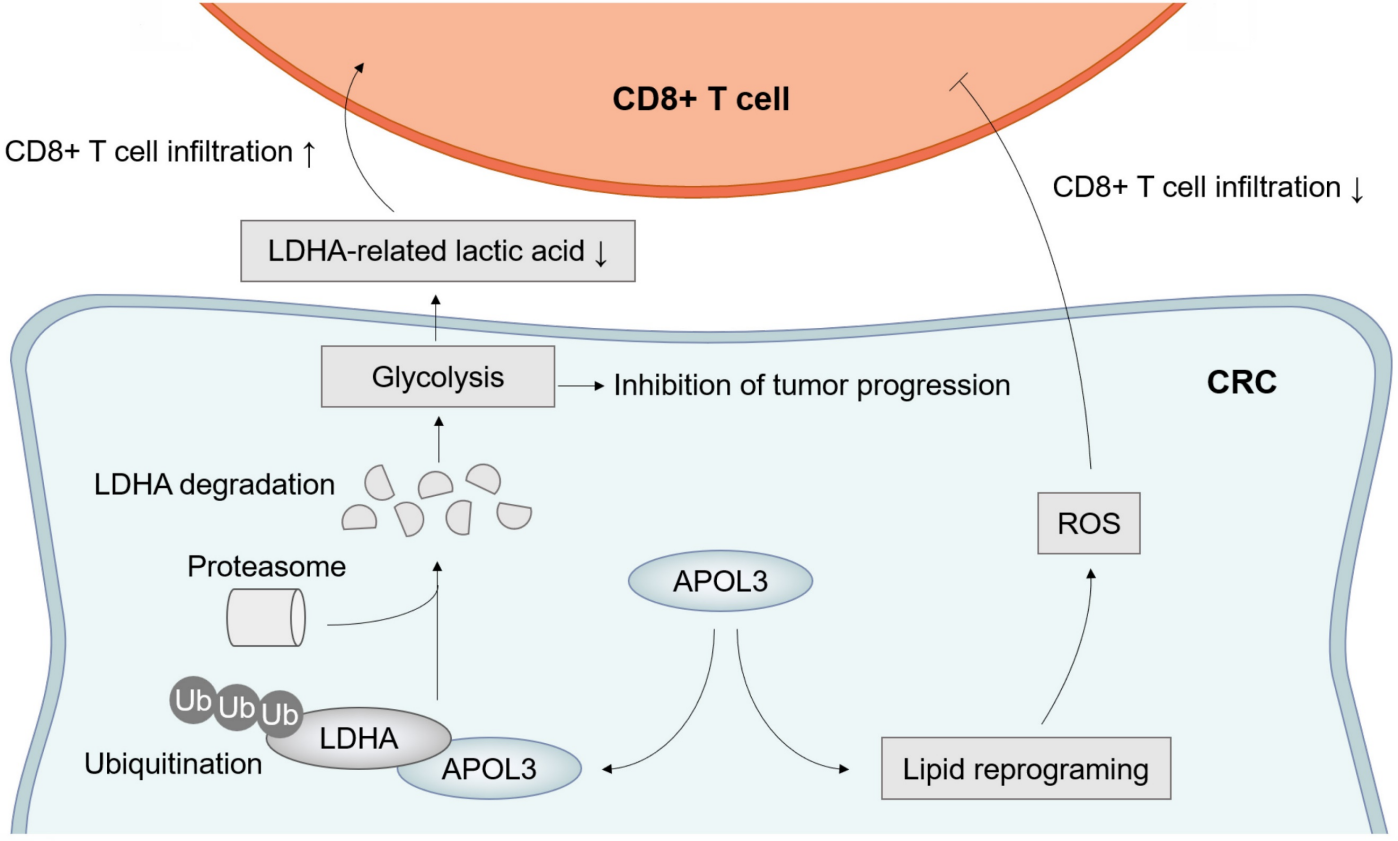
Model of APOL3-LDHA axis in CRC progression, ferroptosis and antitumor immunity. **Abbreviations:** CRC, colorectal cancer.

Taken together, these results further suggested the molecular mechanism by which APOL3 controls tumor proliferation, ferroptosis and anti-tumor immunity via promoting ubiquitylation-related degradation of LDHA in CRC (**Figure 7**).

## Discussion

Ferroptosis has been revealed as a novel anticancer strategy for metabolically eliminating cancer cells 26. In some cases, this metabolic reprogramming has been linked to an acquired sensitivity to ferroptosis and reshaped tumor microenvironment, thus opening up new opportunities to treat immunotherapy-insensitive tumors such as CRC 17. Therefore, a better understanding of the processes that regulate ferroptosis sensitivity should ultimately aid in the discovery of novel therapeutic strategies to improve CRC treatment. In our previous study, based on colorectal tumor microarray, we found that three ferroptosis genes were prognosis-related markers and correlated with immune-activation (paper unpublished), and how modulation of ferroptosis leading to immune-supportive microenvironment is unknown. Here, by performing a three-phase screen (Cox analysis, WGCNA analysis and differential expression analysis), we identified the APOL3 as a novel CRC-related ferroptotic marker and a modulator for CD8+ T cell infiltration.

As an apolipoprotein, APOL3 was reported in many cell types, such as endothelial cells 27, podocyte 28, neurones^29^, trypanosomes^30^. Recently, Gaudet et al. 31 reported that IFN-γ-induced APOL3 was a potent bactericidal agent protecting multiple non-immune barrier cell types against infection. Besides, the phenotype of APOL3^KO^ podocytes exhibits striking similarities to that resulting from cancer metastasis, with strong reduction of cellular adherence and increase in cell motility 27. In cancer, the APOL3-controlled NCS-1 is known to promote motility, metastatic spread and survival of cancer cells 32, 33. Moreover, such phenotype could result from the decrease in GOLPH3 association with trans-Golgi membranes due to the reduction of PI(4)P levels, which play a role in metastasis of breast cancers and glioblastoma^34^. Also, APOLs were reported in several cancers, including cervical, ovarian, breast, thyroid, bladder and prostate cancers 35-37. However, in CRC, little is known about its role in ferroptosis and CD8+ T cells. In this study, we found that APOL3-mediated ferroptosis in CRC enhanced intra-tumoral CD8+ T cell effector function and promoted their antitumor ability.

Before exploring the role of APOL3 in CRC, we performed a in silicon validation using ferroptosis-related heatmap analysis and immune infiltration analysis (including SsGSEA, MCPCOUNTER, CIBERSORT, Xcell and Quantiseq analysis). In the present work, we found that APOL3 was commonly downregulated in CRC, besides, low expression of APOL3 was related to poor survival and was an unfavorable prognostic indicator of CRC patients. To validate the biological function of APOL3 in CRC, we firstly constructed APOL3-knockout and APOL3-overexpression CRC cell lines. Overexpression of APOL3 inhibited cancer cell growth in CRC cells, while knockout of APOL3 promoted cancer cell proliferation. Next, we identified that APOL3 was selectively involved in promotion of RSL3-induced ferroptosis in the CRC cell line. To explore the mechanism, we further performed both transcriptome sequencing and protein-protein interaction network prediction to identify LDHA as the possible downstream molecule of APOL3 in CRC. The following analysis and IP-western assay identified LDHA as the ubiquitylation substrate of APOL3, and knockdown of APOL3 led to elevated protein levels of LDHA, which decreased the sensitivity to RSL3 in CRC cell lines. Overall, abnormal levels of APOL3 appeared to consistently exert tumor suppressor tendencies in CRC cell lines.

In present work, LDHA degradation was proven to be of considerable significance for ferroptosis and tumor progression. The expression of LDHA was increased after induction with RSL3, and CRC cells overexpressing LDHA significantly inhibits RSL3-induced ferroptosis. Effect of LDHA on immunity were explored in many biological models. A previous report showed that LDHA-associated lactic acid production blunts tumor immunosurveillance by CD8+ T cells 25. Besides, Xu et al recently found that ablation of LDHA inhibits PI3K-dependent phosphorylation of Akt and its transcription factor target Foxo1, causing defective antimicrobial immunity 38. Consistently, in our study, by using co-culture and in vivo analysis, we found that APOL3-LDHA axis could directly enhance antitumor ability of effector T cells with higher IFNG expression and lower lactic acid concentration. In addition, as overexpression of APOL3 promoted RSL3-induced ferroptosis through LDHA, we further found that in immunocompetent mice that overexpression of APOL3 could enhance the treatment effect of RSL3 and PDL1 inhibitor. This regulation process can limit the negative feedback induced by ferroptosis inducer and immunotherapy in CRC.

Obviously, there were some limitations in this study. Firstly, the screen was based on the data of the public database, such TCGA and GEO database; however, we performed an integrated screen based on both proteomics and transcriptomics data, we hope the multi-omics screen process could partly solved this limitation. Secondly, the clinical validation was based on IHC staining of serial sections instead of multiplex IHC staining, we believed that large sample test and the combination of IHC and IF can solve this problem.

Finally, clinical relevance of APOL3 with ferroptosis, immunity was also evaluated in CRC specimen. First, based on TCGA data, we demonstrated a significant correlation between APOL3 and cytotoxic cytokine-associated gene expression; next, based on single-cell RNA sequence, we found that APOL3 expression were correlated with tumor microenvironment infiltration; Furthermore, based on IF and IHC analysis, APOL3 was significantly correlated with LDHA expression and CD8+ T cell infiltration.

## Conclusion

In summary, through COX analysis, WGCNA analysis and differential expression analysis, our study illustrates that APOL3 functions as an important factor in CRC. we found APOL3 expression occurred with higher CD8+ T cell in CRC and decreased APOL3 level was strongly associated with malignant properties of CRC cells. Mechanically, APOL3 can promote ubiquitylation-related degradation of LDHA, APOL3-LDHA axis inhibits cancer cell proliferation, promote ferroptosis and CD8+ T cells' antitumor ability. This finding also demonstrates that overexpression of APOL3 may serve as a potential cellular mechanism of sensitivity to RSL3 and immunotherapy. Finally, we found that APOL3 served as a biomarker for assessing treatment effectiveness on ferroptosis-based immunotherapy and an intervention target of CRC (**Figure 7**).

## Supplementary Material

Supplementary materials and methods, figures and tables.

Supplementary datasets.

## Abbreviations

CRC: colorectal cancer
TCGA: the cancer genome atlas
WGCNA: weighted gene co-expression network analysis
CPTAC: Clinical Proteomic Tumor Analysis Consortium
GSEA: Gene Set Enrichment Analysis
ACT: adjuvant chemotherapy
TMA: tissue microarray
IHC: immunohistochemical staining
PLR: positive likelihood ratio
CI: confidence interval
PPI: protein-protein interaction
FC: fold change
NSE: Normalized Enrichment Score
KO: knock-out
WB: Western blotting
qRT-PCR: Reverse transcriptase polymerase chain reactions
IFN-γ: interferon-γ

## References

[1] Siegel RL, Miller KD, Goding Sauer A, Fedewa SA, Butterly LF, Anderson JC (2020). Colorectal cancer statistics, 2020. CA Cancer J Clin.

[2] Siegel RL, Miller KD, Jemal A (2020). Cancer statistics, 2020. CA Cancer J Clin.

[3] Oliphant R, Nicholson GA, Horgan PG, Molloy RG, McMillan DC, Morrison DS (2013). Contribution of surgical specialization to improved colorectal cancer survival. Br J Surg.

[4] Yuan Y, Jian J, Jing H, Yan R, You F, Fu X (2021). Single-Incision vs. Conventional Laparoscopic Surgery for Colorectal Cancer: An Update of a Systematic Review and Meta-Analysis. Front Surg.

[5] Chen W, Zheng R, Baade PD, Zhang S, Zeng H, Bray F (2016). Cancer statistics in China, 2015. CA Cancer J Clin.

[6] Andre T, Amonkar M, Norquist JM, Shiu KK, Kim TW, Jensen BV (2021). Health-related quality of life in patients with microsatellite instability-high or mismatch repair deficient metastatic colorectal cancer treated with first-line pembrolizumab versus chemotherapy (KEYNOTE-177): an open-label, randomised, phase 3 trial. Lancet Oncol.

[7] Andre T, Shiu KK, Kim TW, Jensen BV, Jensen LH, Punt C (2020). Pembrolizumab in Microsatellite-Instability-High Advanced Colorectal Cancer. N Engl J Med.

[8] Beatty GL, Winograd R, Evans RA, Long KB, Luque SL, Lee JW (2015). Exclusion of T Cells From Pancreatic Carcinomas in Mice Is Regulated by Ly6C(low) F4/80(+) Extratumoral Macrophages. Gastroenterology.

[9] Joyce JA, Fearon DT (2015). T cell exclusion, immune privilege, and the tumor microenvironment. Science.

[10] Sprooten J, De Wijngaert P, Vanmeerbeerk I, Martin S, Vangheluwe P, Schlenner S (2020). Necroptosis in Immuno-Oncology and Cancer Immunotherapy. Cells.

[11] Galluzzi L, Garg AD (2021). Immunology of Cell Death in Cancer Immunotherapy. Cells.

[12] Dixon SJ (2017). Ferroptosis: bug or feature?. Immunol Rev.

[13] Zuo T, Fang T, Zhang J, Yang J, Xu R, Wang Z pH-Sensitive Molecular-Switch-Containing Polymer Nanoparticle for Breast Cancer Therapy with Ferritinophagy-Cascade Ferroptosis and Tumor Immune Activation. Adv Healthc Mater. 2021: e2100683.

[14] Cai S, Fu S, Zhang W, Yuan X, Cheng Y, Fang J (2021). SIRT6 silencing overcomes resistance to sorafenib by promoting ferroptosis in gastric cancer. Biochem Biophys Res Commun.

[15] Lu Y, Qin H, Jiang B, Lu W, Hao J, Cao W (2021). KLF2 inhibits cancer cell migration and invasion by regulating ferroptosis through GPX4 in clear cell renal cell carcinoma. Cancer Lett.

[16] Yadav P, Sharma P, Sundaram S, Venkatraman G, Bera AK, Karunagaran D (2021). SLC7A11/ xCT is a target of miR-5096 and its restoration partially rescues miR-5096-mediated ferroptosis and anti-tumor effects in human breast cancer cells. Cancer Lett.

[17] Friedmann Angeli JP, Krysko DV, Conrad M (2019). Ferroptosis at the crossroads of cancer-acquired drug resistance and immune evasion. Nat Rev Cancer.

[18] Lv Y, Feng QY, Zhang ZY, Zheng P, Zhu DX, Lin Q (2022). Low ferroptosis score predicts chemotherapy responsiveness and immune-activation in colorectal cancer. Cancer Med.

[19] Becht E, Giraldo NA, Lacroix L, Buttard B, Elarouci N, Petitprez F (2016). Estimating the population abundance of tissue-infiltrating immune and stromal cell populations using gene expression. Genome Biol.

[20] Newman AM, Liu CL, Green MR, Gentles AJ, Feng W, Xu Y (2015). Robust enumeration of cell subsets from tissue expression profiles. Nat Methods.

[21] Aran D, Hu Z, Butte AJ (2017). xCell: digitally portraying the tissue cellular heterogeneity landscape. Genome Biol.

[22] Finotello F, Mayer C, Plattner C, Laschober G, Rieder D, Hackl H (2019). Molecular and pharmacological modulators of the tumor immune contexture revealed by deconvolution of RNA-seq data. Genome Med.

[23] Warde-Farley D, Donaldson SL, Comes O, Zuberi K, Badrawi R, Chao P (2010). The GeneMANIA prediction server: biological network integration for gene prioritization and predicting gene function. Nucleic Acids Res.

[24] Patil A, Nakai K, Nakamura H (2011). HitPredict: a database of quality assessed protein-protein interactions in nine species. Nucleic Acids Res.

[25] Brand A, Singer K, Koehl GE, Kolitzus M, Schoenhammer G, Thiel A (2016). LDHA-Associated Lactic Acid Production Blunts Tumor Immunosurveillance by T and NK Cells. Cell Metab.

[26] Hassannia B, Vandenabeele P, Vanden Berghe T (2019). Targeting Ferroptosis to Iron Out Cancer. Cancer Cell.

[27] Khalil A, Poelvoorde P, Fayyad-Kazan M, Rousseau A, Nuyens V, Uzureau S (2018). Apoliporotein L3 interferes with endothelial tube formation via regulation of ERK1/2, FAK and Akt signaling pathway. Atherosclerosis.

[28] Uzureau S, Lecordier L, Uzureau P, Hennig D, Graversen JH, Homble F (2020). APOL1 C-Terminal Variants May Trigger Kidney Disease through Interference with APOL3 Control of Actomyosin. Cell Rep.

[29] Boeckel GR, Ehrlich BE (2018). NCS-1 is a regulator of calcium signaling in health and disease. Biochim Biophys Acta Mol Cell Res.

[30] Fontaine F, Lecordier L, Vanwalleghem G, Uzureau P, Van Reet N, Fontaine M (2017). APOLs with low pH dependence can kill all African trypanosomes. Nat Microbiol.

[31] Gaudet RG, Zhu S, Halder A, Kim BH, Bradfield CJ, Huang S (2021). A human apolipoprotein L with detergent-like activity kills intracellular pathogens. Science.

[32] Apasu JE, Schuette D, LaRanger R, Steinle JA, Nguyen LD, Grosshans HK (2019). Neuronal calcium sensor 1 (NCS1) promotes motility and metastatic spread of breast cancer cells in vitro and in vivo. FASEB J.

[33] Grosshans HK, Fischer TT, Steinle JA, Brill AL, Ehrlich BE (2020). Neuronal Calcium Sensor 1 is up-regulated in response to stress to promote cell survival and motility in cancer cells. Mol Oncol.

[34] Sechi S, Frappaolo A, Karimpour-Ghahnavieh A, Piergentili R, Giansanti MG (2020). Oncogenic Roles of GOLPH3 in the Physiopathology of Cancer. Int J Mol Sci.

[35] Chidiac M, Fayyad-Kazan M, Daher J, Poelvoorde P, Bar I, Maenhaut C (2016). ApolipoproteinL1 is expressed in papillary thyroid carcinomas. Pathol Res Pract.

[36] Johanneson B, McDonnell SK, Karyadi DM, Quignon P, McIntosh L, Riska SM (2010). Family-based association analysis of 42 hereditary prostate cancer families identifies the Apolipoprotein L3 region on chromosome 22q12 as a risk locus. Hum Mol Genet.

[37] Hu CA, Klopfer EI, Ray PE (2012). Human apolipoprotein L1 (ApoL1) in cancer and chronic kidney disease. FEBS Lett.

[38] Xu K, Yin N, Peng M, Stamatiades EG, Shyu A, Li P (2021). Glycolysis fuels phosphoinositide 3-kinase signaling to bolster T cell immunity. Science.

