# Supplementary material for: Apolipoprotein L3 enhances CD8+ T cell antitumor immunity of colorectal cancer by promoting LDHA-mediated ferroptosis
Source: Int J Biol Sci. 2023 Feb 13;19(4):1284–98. doi: 10.7150/ijbs.74985 (PMC10008698; doi:10.7150/ijbs.74985)
Supplement: Supplementary file 1 — Supplementary materials and methods, figures and tables. [file ijbsv19p1284s1.pdf]

Supplementary information For

Apolipoprotein L3 enhances CD8+ T cell antitumor immunity of colorectal cancer by promoting LDHA-mediated ferroptosis

Authors: Lv Yang[1,2#], Tang WenTao[1,2,3#], Zhang ZhiYuan[1,2,3#], Chang WenJu[1,2,3#], Lin Qi[1,2,3], Ji MeiLing[1,2,3], Xu YuQiu[1,2,3], Feng QingYang[1,2,3], He GuoDong[1,2,3*], Xu JianMin[1,3,4*]

Corresponding authors: Xu JianMin, E-mail: xujmin@aliyun.com and Guo-Dong He, E-mail: angelhgd@163.com

This PDF file includes:

1. Supplementary Materials and Methods
2. Tables S1 to S4
3. Figures. S1 to S13

Other supplementary materials for this manuscript include the following (not in this file):

Datasets S1 to S9

Supplementary information

Contents

1. **Supplementary methods and materials**
2. **Supplementary tables**

   Table S1: ferroptosis related gene according to publications

   Table S2: Clinical correlation between APOL3 expression level and clinical characteristics in CRC patients

   Table S3: Small guide RNA sequencing

   Table S4: Primers for q-PCR assay
3. **Supplementary Figures' legends**

   Figure S1: GO analysis for validation of WGCNA modules in Blue (A), Green (B), Red (C), Yellow (D), Brown (E), Black (F), Turquoise (G) and Grey (H) cohorts. Abbreviation: GO, gene ontology; WGCNA, Weighted Gene Co-expression Network Analysis.

   Figure S2: differential analysis of ACLS4 (A), GPX4 (B) and NOX1 (C) based on TCGA CRC transcriptome data; (D) overlapping the differential analysis and the pink gene list, APOL3 and APOL6 were screened. Abbreviation: TCGA, the cancer genome atlas; CRC, colorectal cancer.

   Figure S3: SsGSEA analysis demonstrated the immune-activation role of APOL3 in CRC. Abbreviation: SsGSEA, single sample gene set enrichment analysis; CRC, colorectal cancer.

   Figure S4: In silicon estimation of APOL3 expression on CD8+ T cell infiltration in CRC specimen. The estimation algorithm included MCPCOUNTER (A), CIBERSORT (B), XCELL (C) and QUANTISEQ (D). Abbreviation: CRC, colorectal cancer.

   Figure S5: mRNA expression correlation between APOLs family genes and ferroptosis-related markers from TCGA database. (A) to (D) separately represented APOL1, APOL2, APOL4 and APOL6. Abbreviation: TCGA, the cancer genome atlas.

   Figure S6: (A) compared to normal control, APOL3 in colon cancer were significantly down-regulated; (B) low expression of APOL3 statistically correlated with higher grade of lymph node metastasis; (C) low expression of APOL3 statistically correlated with higher grade of distant metastasis.

   Figure S7: (A) Baseline mRNA expression of APOL3 in CRC cell line HT29, HCT116, CACO2 and RKO; (B) Baseline WB analysis for APOL3 in CRC cell line HT29, HCT116, CACO2 and RKO; (C) construction of APOL3-knock out in RKO and HCT116 cells, and the relative APOL3 mRNA expression to GAPDH was analyzed by qRT-PCR; (D) compared to control, WB analysis for APOL3 was decreased in APOL3-Sg1 and APOL3-Sg2 cells. Abbreviation: WB, western blotting; qRT-PCR, Quantitative Real-time polymerase chain reaction.

   Figure S8: (A) Construction of APOL3-OE in HT29 and CACO2 cells, and the relative APOL3 mRNA expression to GAPDH was analyzed by qRT-PCR; (B) compared to control, WB analysis for APOL3 was decreased in APOL3-OE cells. Abbreviation: WB, western blotting; qRT-PCR, Quantitative Real-time polymerase chain reaction.

   Figure S9: subcutaneous xenograft models of APOL3-overexpressed HT29 CRC cells were constructed. (A) tumor volume lines were drawn to compared the different cohorts, overexpression of APOL3 significantly inhibits HT29 tumor growth in vivo; (B) tumor weight was also compared among different cohorts. Abbreviation: CRC, colorectal cancer.

   Figure S10: subcutaneous xenograft models of APOL3-overexpressed HT29 CRC cells were constructed and treated by RSL3. (A) tumor volume lines were drawn to compared the different cohorts, overexpression of APOL3 significantly promoted HT29 cell death in vivo; (B) tumor weight was also compared among different cohorts. Abbreviation: CRC, colorectal cancer.

   Figure S11: relative Fe2+ concentration (A) and MDA (%) (B) in subcutaneous xenograft models of HT29 cells.

   Figure S12: relative Fe2+ concentration (A) and MDA (%) (B) in subcutaneous xenograft models of HT29 cells treated by RSL3.

   Figure S13: external single-cell RNA sequence validation for APOL3 on CRC

microenvironment from public dataset (https://omnibrowser.abiosciences.com/). Abbreviation: CRC, colorectal cancer.

Supplementary Method and materials

CPTAC, TCGA data Source, Processing and differential analysis

Raw data of RNA sequence and matched clinical characteristics of colon and rectal cancer were downloaded from the online database The Cancer Genome Atlas (TCGA): https://tcga-data.nci.nih.gov/tcga/. CPTAC data were retrieved from https://proteomics.cancer.gov/programs/cptac. The cohort contains 417 tumor tissues. Next, we performed gene differential analysis (|LogFC| > 0.5, adjusted P value (FDR) < 0.05) by comparing tumor tissues using limma R package based on ACSL4, NOX1 and GPX4. Cut-off value of ACSL4, NOX1 and GPX4 mRNA expression and protein level were determined by medians. The integrated up-regulated and dysregulated gene lists were saved for subsequent analysis.

Cell culture

Immortalized human HEK293T (ATCC catalog #ACS-4500), CRC cell lines (ATCC catalog #CRL-1997), HCT116 (ATCC catalog #CCL-247), Caco2 (ATCC catalog #HTB-37), HT29(ATCC catalog #HTB-38) and RKO (ATCC catalog #FS-0347) were purchased from American Type Culture Collection (ATCC). HEK293T and SW1463 was maintained in RPMI 1640 (Thermo Fisher Scientific #11875-093) medium containing 10% FBS and penicillin/streptomycin. HCT116, Caco2, HT29 and RKO were maintained in Dulbecco's modified Eagle's medium (Thermo Fisher Scientific #11320) medium with 10% FBS and penicillin/streptomycin. All cells were cultured in a 5% CO2 humidified atmosphere at 37°C.

Plasmid constructs and lentivirus production

The empty vector (EV) was purchased from GeneCopoeiaTM (#EX-Z2866-Lv105). Short guide RNAs (sgRNAs) that target human APOL1, APOL2, APOL3, APOL4, APOL6 and LDHA were designed using the Optimized CRISPR Design web tool (http://crispr.mit.edu/). APOL3 sgRNA#1 (Sg1) and sgRNA#2 (Sg2) vectors were generated by cloning these two APOL3 targeting sgRNAs into the lentiCRISPRv2 vector (Addgene, plasmid #52961) individually. SgRandom control was constructed by cloning a sgRNA with a random sequence into the lentiCRISPRv2 vector. Lentivirus particles were generated by co-transfecting these lentiviral constructs with helper virus packaging plasmids pCMVΔR8.9 and pHCMV-VSV-G into HEK293T cells using Lipofectamine 3000 (Invitrogen# L3000015). Lentiviruses were harvested after 24, 48 and 72 h, and frozen at –80°C in aliquots at appropriate amounts for infection. Small guide RNA sequence was listed in Table S3.

Real-time quantitative RT-PCR and quantitative PCR

Tissues were homogenized in TRIzol Reagent (Invitrogen; #15596026), followed by total RNA isolation using the standard protocol. The RNA was further reverse-transcribed into cDNA using the TransScript First-Strand cDNA Synthesis SuperMix (TransGen; #AT301). Genomic DNA was isolated using QIAamp DNA Mini Kit (QIAGEN; #51306). qPCR was performed for target-gene-expression analysis or exon detection of CUL9 using the iQTM SYBR Green Supermix (Bio-Rad; #170-8882). Samples were run in triplicate with non-reverse transcriptase or non-template control. Amplification accuracy was verified by melting curve analysis. Relative mRNA expression was normalized to GAPDH expression as an internal amplification control. Specific qPCR primers are listed in Table S4.

Western Blot analysis

Whole, nuclear and cytoplasmic cell lysates from cell lines were prepared using lysis buffer (1% NP-40, 50 mM Tris-HCl pH 8.0, 100 mM sodium fluoride, 30 mM sodium pyrophosphate, 2 mM sodium molybdate, 5mM EDTA, 2 mM sodium orthovanadate) containing or not containing protease inhibitors. The lysates were then rocked overnight at 4°C and cleared by centrifugation at 14,000rpm for 30min at 4°C. The lysate protein concentrations were determined using a Quick StartTM Bradford 1× Dye Reagent (Bio-Rad; #5000205). Electrophoresis and western blotting were performed using standard techniques. The hybridization signals were detected by chemiluminescence (Immobilon Western, Millipore Corporation, MA) and captured using an Amersham Imager 600 imagers (GE Healthcare; #29083461). Cells were grown until 80% confluence, washed with PBS and harvested. Primary

antibodies were APOL3 (abcam#ab154869), APOL6 (abcam#ab92273), GPX4 (abcam#125066), NOX1 (abcam#131088), FACL4 (abcam#155282), LDHA (CST#3582), c-PARP (CST#9548), PCNA (Santa Cruz#sc-56), p-MAPK (CST#9101), MAPK (CST#9102), p-AKT (CST#9271), AKT (CST#9272), GAPDH (Sigma#G8795), p21 (CST#2941), FTH1 (CST#4393), COX-2 (CST#12282), HK2 (#CST2867), β-Actin (CST#3700).

Malondialdehyde (MDA) and Iron assay

MDA concentration in cell lysates was assessed using a lipid peroxidation assay kit (abcam#118970) from Abcam according to the manufacturer's instructions. Cells were seeded in 10 cm plate (5× 106 cells per plate) and treated with DMSO or RSL3 for 24 h. Cells were slightly washed with ice-cold PBS and homogenized on ice in 300 µl of the MDA lysis buffer with 3 µl BHT (100×), then centrifuged (13,000 × g, 10 min) to remove insoluble material. Place 200 µl of the supernatant from each homogenized sample into a microcentrifuge tube. Add 600 µL of the thiobarbituric acid (TBA) solution into each vial. Incubate samples at 95 °C for 60 min. The MDA in the sample reacted with TBA to generate an MDA-TBA adduct. Cool samples to room temperature in an ice bath for 10 min. Pipette 200 mL from each reaction mixture into a 96-well plate for analysis. Measure the absorbance at 532 nm using a microplate reader.

Intracellular ferrous iron (Fe2+) level was determined using the iron assay kit (abcam#83366) purchased from Abcam according to manufacturer's instructions. Cells were seeded onto 10 cm plate (5 × 106 cells per plate) and treated with DMSO or erastin for 24 h. Cells were collected and washed in ice-cold PBS and homogenized in 5× volumes of iron assay buffer on ice, then centrifuged (13,000 × g, 10 min) at 4 °C to remove insoluble material. Collect the supernatant and add iron reducer to each sample, mix, and incubate for 30 min at room temperature. Add 100 µl of iron probe to each sample. Mix well using a horizontal shaker or by pipetting, and incubate the reaction for 60 min at room temperature. Protect the plate from light during the incubation. Measure the absorbance at 593-nm using a microplate reader.

Colony formation assay

Six-well plates were first layered with 0.6% bottom agar (Noble agar, BD difco #214220) containing RPMI 1640 medium with 10% FBS and penicillin/streptomycin. HCT116-empty vector ($2X10^5$/per well), HCT116-APOL3 Sg1 ($2X10^5$ per well) and HCT116-APOL3 Sg2 ($2X10^5$ per well) were seeded in 0.4% top agar containing 10% FBS and penicillin/streptomycin. Cells were allowed to grow for 3-5 weeks and then stained with 1 ml of 1 mg/ml methyl thiazol tetrazolium (Sigma–Aldrich #M5655) for 3 h. Colonies were counted manually, and the clone size was measured by Image-J software (National Institutes of Health). All assays were performed in triplicate wells, with the entire study replicated at least once. Clone formation assay was conducted by seeding CRC cells in different cohorts (1000 per well) cells into six-well plates and allowed to grow for 3 weeks. Then, the cells were fixed with 4% paraformaldehyde for 15 min and stained with crystal violet solution (Wuhan Servicebio Technology Co. #G1014) for 3 h. All assays were carried out in triplicate wells, with the entire study replicated at least once. Images were obtained using a scanner (Microtek, TMA 1600III).

Cell viability assays and analysis.

Cell Viability studies were performed using the CellTiter-Glo luminescent assay (Promega, Madison, WI). Cells were plated at 5,000-20,000 cells per well in a 96-well flat-bottomed plate (Falcon, Lincoln, NJ). Luminescence was analyzed using a Veritas microplate luminometer (Turner Biosystems, Sunnyvale, CA).

Immunohistochemistry and intensity evaluation

Formalin-fixed paraffin-embedded surgical specimens were used for tissue microarray (TMA) construction and subsequent immunohistochemistry (IHC) study as described previously. Histological review was also conducted to avoid necrotic and hemorrhagic tumor regions. The immunoreactivity for APOL3 (abcam#ab154869), APOL6 (abcam#ab92273), LDHA (CST#3582), HK2 (#CST2867), GPX4 (abcam#125066), NOX1 (abcam#131088) and FACL4 (abcam#155282) in cancer cells was calculated as the product of two independent scores, the proportion of positive tumor cells in the tissues and the average intensity of positive tumor cells in the tumor tissues. The expression was scored

independently by two pathologists who were blinded to clinical pathological characteristics. Cut-off was determined as median score.

Multicolour immunohistochemistry and immunofluorescence analysis

The TMAs of the CRC specimen were constructed as previously described. Dual immunohistochemistry (IHC) staining was performed. Briefly, the TMA slides were dewaxed in an oven and treated with water bath-heated xylene and graded alcohols. The slides were heated in sodium citrate buffer (0.01 M sodium citrate buffer, pH = 6) for 15 min for antigen retrieval. Normal goat serum blocking solution was applied for 20 min at 37 °C. Then, anti-APOL3 antibody (Abcam, ab154869, diluted at 1:300) was applied for 2 h at 37 °C. After the primary antibody incubation, a general two-step kit detection system was used (HRP, Mo/Ra; ZSGB Biotech, PV-9000) with DAB. The slides were washed again and incubated with anti-CD8A primary antibody (Abcam, ab199016, diluted at 1:1500) overnight at 4 °C. TMA slides were subsequently washed, incubated with AP-labelled secondary antibody and stained with Vector Blue (Vector Blue AP Substrate Kit detection system; Vector Labs, SK-5300). Finally, the sections were washed, dehydrated and mounted. For immunofluorescence staining, the sections were incubated with the primary antibodies overnight at 4 °C. Then, samples were incubated with FITC-conjugated and TRITC-conjugated secondary antibodies for 2 h at 37 °C. Finally, the slides were mounted with anti-fade mounting solution containing DAPI. The slides were captured using a Leica DMi8 microscope.

Animal analysis

The animal experiments were approved by Institutional Animal Care and Use Committee (IACUC) of the Shanghai Institutes for Biological Sciences, Chinese Academy of Sciences. Inclusion criteria were as follows: 1) body weights must be within a certain range for the tolerance of subcutaneous injection; 2) adaptability for the environment of animal feeding room. Exclusion criteria were: 1) death within 1 week after injection; 2) unintended death during experiment, such as Cachexia; 3) unmeasurable tumor volume. For in vivo proliferation evaluation, 2 experimental units were included. Unit 1: HCT-116 controls (empty vector) and APOL3-KO colon cancer cells (2×10^6) in PBS were injected subcutaneously into 6-week-old female BALB/c nude mice. Unit 2: HCT-116 controls (empty vector) and APOL3-KO colon cancer cells (2×10^6) in PBS were injected subcutaneously into 6-week-old female BALB/c nude mice treated by RSL3. Unit 3: HT29 controls (empty vector) and APOL3-OE colon cancer cells (4×10^6) in PBS were injected subcutaneously into 4- week-old female BALB/c nude mice. Unit 4: HT29 controls (empty vector) and APOL3-OE colon cancer cells (4×10^6) in PBS were injected subcutaneously into 4-week-old female BALB/c nude mice treated by RSL3. Unit 5: CT26 controls (empty vector) and APOL3-KO CT26 cells (4×10^6) in PBS were injected subcutaneously into 4-week-old female BALB/c nude mice. Unit 6: CT26 controls (empty vector) and APOL3-KO CT26 cells (4×10^6) in PBS were injected subcutaneously into 4-week-old female BALB/c immune-competent mice. The resulting tumors were measured every 6 days. Tumor volume was calculated using the formula: tumor volume= length×width×height/2. Once the largest tumor diameter reached the maximal tumor diameter allowed under our institutional protocol, all mice were killed, and tumors were collected. The maximal tumor diameter allowed by the IACUC is 2.0 cm.

For in vivo agent assay, BALB/c mice were treated with RSL3 (30 mg/kg, intraperitoneal injection, daily for 2 weeks) or PD1 (atezolizumab, 50 mg/kg, intraperitoneal injection daily for 3 days) as single agent or in combination. The resulting tumors were measured every 6 days. Tumor volume was calculated using the formula: tumor volume = length×width×height/2. Once the largest tumor diameter reached the maximal tumor diameter allowed under our institutional protocol, all mice were killed, and tumors were collected. The maximal tumor diameter allowed by the IACUC is 2.0 cm.

Statistical analysis was performed using GraphPad Prism 6 (GraphPad Software, La Jolla, CA, USA). The difference between two groups was analyzed by paired sample t-test. The differences among multiple groups were analyzed by one-way analysis. Analysis of the animal data were performed blindly by an independent author without the knowledge of group information.

Supplementary tables

Table S1: ferroptosis related gene according to publications

NCOA4 ACO1 FTH1 STEAP3 FANCD2 NFS1 TFRC PHKG2 IREB2 HSBP1 HMOX1 CISD1/mitoNEET.

ACSF2 CS(citrate synthase) LPCAT3 ACSL4 ACSL3 ACACA GPX4 AKR1C LOX PEBP1 ZEB1 SQS/FDFT1 SQLE HMGCR FADS2

SLC1A5 GLS2 GOT1 G6PD PGD

NRF2 KEAP1 HMOX1 NQO1 SLC7A11 GCLC CARS CBS NOX1 ABCC1/MRP1

Table S2: Clinical correlation between APOL3 expression level and clinical characteristics in CRC patients

	Cases	APOL3		
**Factors**		**Low (%)**	**High (%)**	**P**
**All patients**	150	69	81	
**Age**				0.963
≤60	91 (60.7)	42 (60.9)	49 (60.5)	
>60	59 (39.3)	27 (39.1)	32 (39.5)	
**Gender**				0.941
Male	93 (62.0)	43 (62.3)	50 (61.7)	
Female	57 (38.0)	26 (45.6)	31 (38.3)	
**CEA (ng/ml)**				0.749
≤ 5	79 (52.7)	29 (42.0)	36 (44.4)	
> 5	65 (43.3)	38 (55.1)	41 (50.6)	
Unknown	6 (4.0)	2 (2.9)	4 (4.9)	
**Maximum tumor size**				0.099
≤4.0 cm	66 (44.6)	40 (58.0)	36 (44.4)	
>4.0 cm	84 (55.4)	29 (42.0)	45 (55.6)	
**Differentiation level**				0.804
Good/Moderate	108 (72.0)	49 (71.0)	59 (72.8)	
Poor/Undifferentiated	42 (28.0)	20 (29.0)	22 (27.2)	
**Histological type**				0.668
Non-mucinous	126 (84.0)	**57 (82.6)**	**69 (85.2)**	
Mucinous	24 (16.0)	12 (17.4)	12 (14.8)	
**Tumor location**				0.322
Right-sided colon	49 (32.7)	22 (31.9)	27 (33.3)	
Left-sided colon	29 (19.3)	10 (14.5)	19 (23.5)	
Rectum	72 (48.0)	37 (53.6)	35 (43.2)	
**T stage**				**0.011**
T1/T2	31 (20.7)	8 (11.6)	23 (28.4)	
T3/T4	119 (79.3)	61 (88.4)	58 (71.6)	

**N stage**				0.453
N0	82 (54.6)	40 (58.0)	42 (51.9)	
N1/N2	68 (45.4)	29 (42.0)	39 (48.1)	
**M stage**				0.710
M0	113 (75.3)	51 (73.9)	62 (76.5)	
M1	37 (24.7)	18 (26.1)	19 (23.5)	

Table S3: Small guide RNA sequencing

SgRNA	Sequence (5')	Sequence (3')
APOL3 Sg1	CACCGCAAGGATGGGACTGGGCCAA	AAACTTGGCCCAGTCCCATCCTTGC
APOL3 Sg2	CACCGCTGGAAGAGATTCGTGACTG	AAACCAGTCACGAATCTCTTCCAGC
LDHA	CACCGTTTCCGCCCACCTTTCCGAG	AAACCTCGGAAAGGTGGGCGGAAAC
APOL1	CACCGTTTTGTCAATATCGTCTGCG	AAACCGCAGACGATATTGACAAAAC
APOL2	CACCGCCAGACCGTCTGATAGCTGG	AAACCCAGCTATCAGACGGTCTGGC
APOL4	CACCGAGGCGTCATAAGCTTCCCGC	AAACGCGGGAAGCTTATGACGCCTC
APOL6	CACCGCAGCATCGTGAGTGGTACGT	AAACACGTACCACTCACGATGCTGC

Table S4: Primers for q-PCR assay

Gene	Sequence (F)	Sequence (R)
APOL3	GACTGCGGCTGAATTGCCCAGGGAT	GCTTCTGCTGATGATGTGTATGAGTG
APOL6	TGGTGTTGGTTTGCAAAGGGAT	TTGGAGCGTTCCAACGTACCACTC
GPX4	GCAACCAGTTTGGGAGGCAGGAG	CCTCCATGGGACCATAGCGCTTC
NOX1	GTTTTACCGCTCCCAGCAGAA	GGATGCCATTCCAGGAGAGAG
ACSL4	AACCCAGAAAACTTGGGCATT	GTCGGCCAGTAGAACCACT
LDHA	CATTGAAGCAAACAATAAGTAGAGG	TGGAGCACATAGCTCAGGTTT
GADPH	GTCTCCTCTGACTTCAACAGCG	ACCACCCTGTTGCTGTAGCCAA
PKM1	CTGAAGGCAGTGATGTGGCC	ACCCGGAGGTCCACGTCCTC
PKM2	ACTCGGGCTGAAGGCAGTGA	TGTGGGGTCGCTGGTAATGG
HK2	TGAGAACTGTCCTTACGTGACC	AGAGCACCAAGACTGGCTCT

Supplementary figures

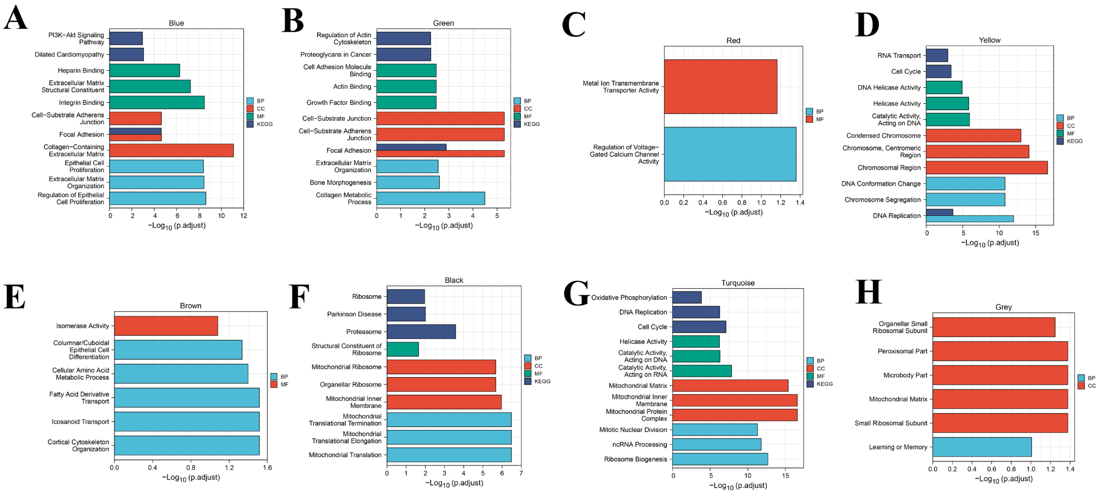

Figure S1: GO analysis for validation of WGCNA modules in Blue **(A)** Green **(B)** Red **(C)** Yellow **(D)** Brown **(E)** Black **(F)** Turquoise **(G)** and Grey **(H)** cohorts. **Abbreviation:** GO gene ontology; WGCNA Weighted Gene Co-expression Network Analysis.

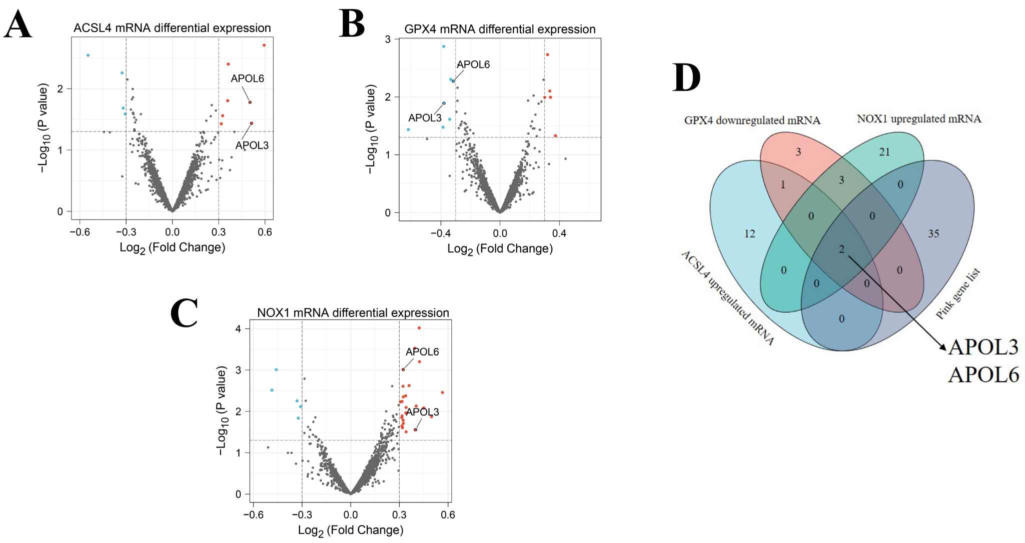

Figure S2: differential analysis of ACLS4 **(A)** GPX4 **(B)** and NOX1 **(C)** based on TCGA CRC transcriptome data; **(D)** overlapping the differential analysis and the pink gene list APOL3 and APOL6 were screened. **Abbreviation:** TCGA the cancer genome atlas; CRC colorectal cancer.

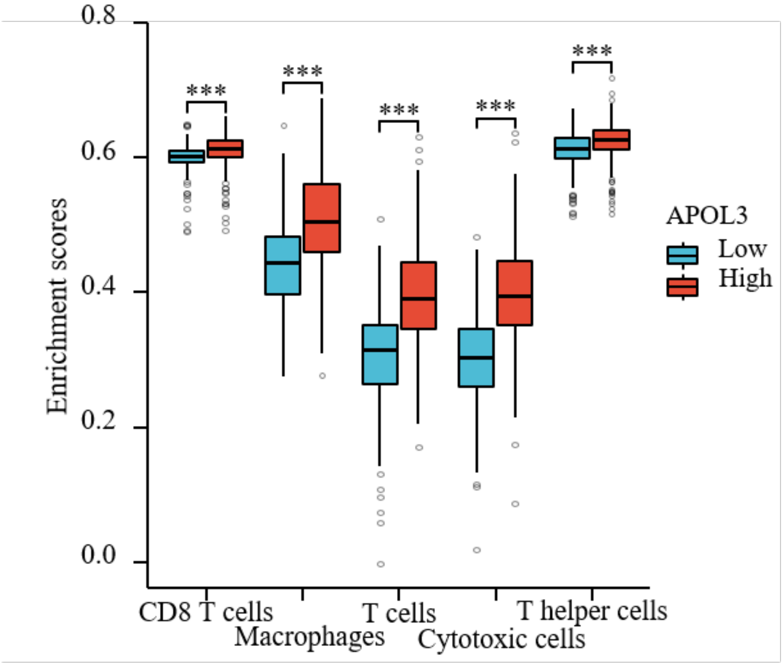

Figure S3: SsGSEA analysis demonstrated the immune-activation role of APOL3 in CRC. **Abbreviation:** SsGSEA single sample gene set enrichment analysis; CRC colorectal cancer.

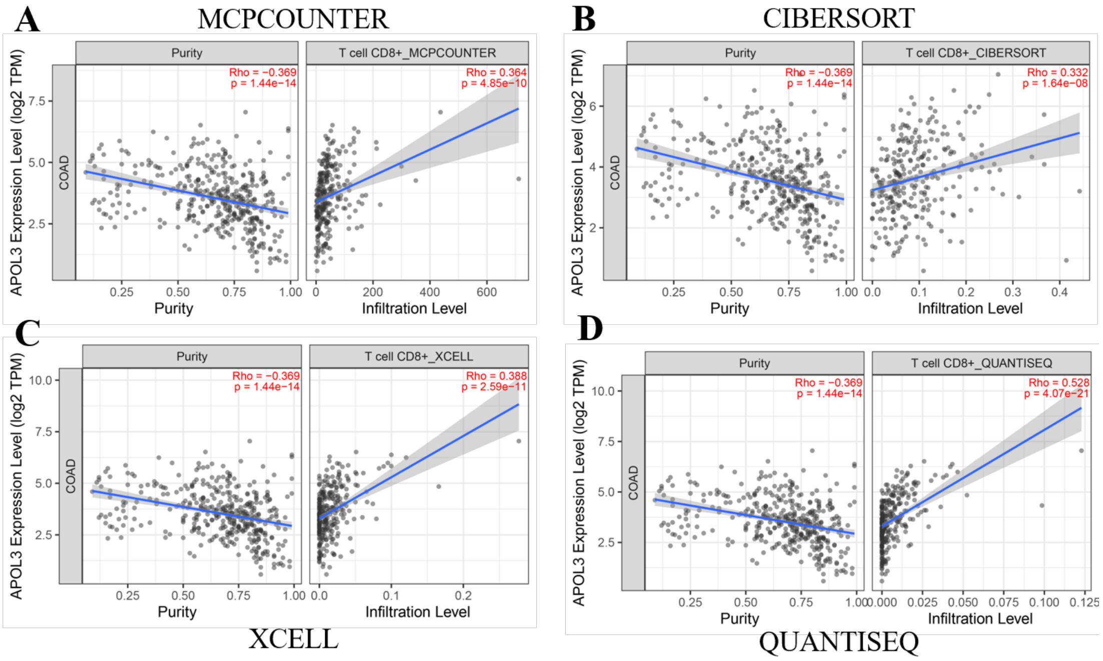

Figure S4: In silicon estimation of APOL3 expression on CD8+ T cell infiltration in CRC specimen. The estimation algorithm included MCPCOUNTER (A) CIBERSORT (B) XCELL (C) and QUANTISEQ (D). **Abbreviation:** CRC colorectal cancer.

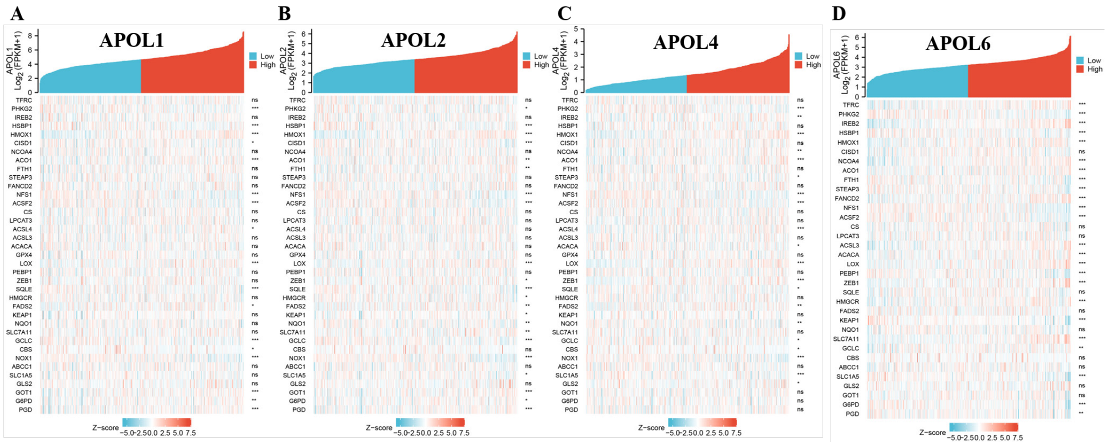

Figure S5: mRNA expression correlation between APOLs family genes and ferroptosis-related markers from TCGA database. (A) to (D) separately represented APOL1 APOL2 APOL4 and APOL6. **Abbreviation:** TCGA the cancer genome atlas.

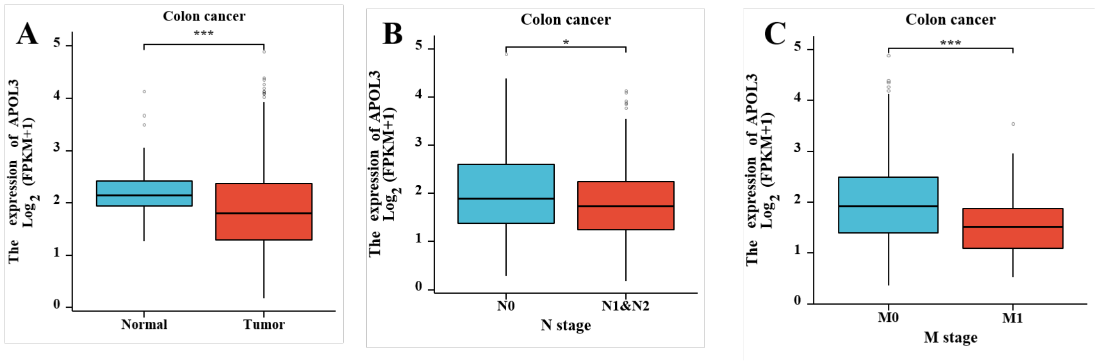

Figure S6: (A) compared to normal control APOL3 in colon cancer were significantly down-regulated; **(B)** low expression of APOL3 statistically correlated with higher grade of lymph node metastasis; **(C)** low expression of APOL3 statistically correlated with higher grade of distant metastasis.

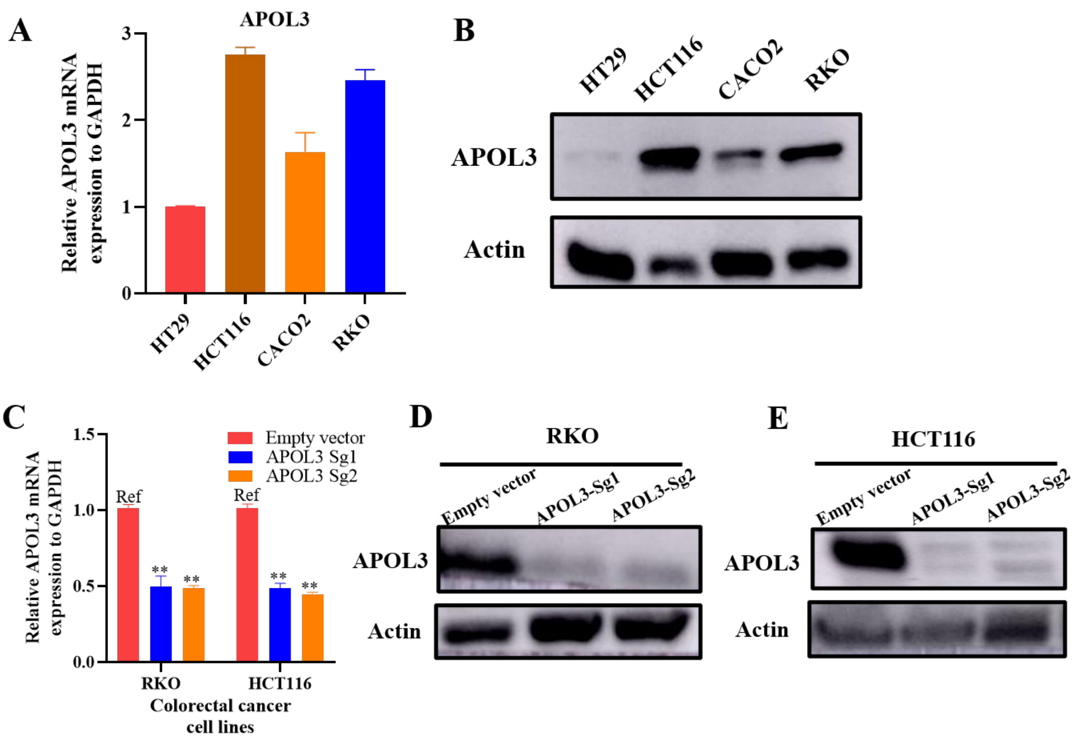

Figure S7: (A) Baseline mRNA expression of APOL3 in CRC cell line HT29 HCT116 CACO2 and RKO; **(B)** Baseline WB analysis for APOL3 in CRC cell line HT29 HCT116 CACO2 and RKO; **(C)** construction of APOL3-knock out in RKO and HCT116 cells and the relative APOL3 mRNA expression to GAPDH was analyzed by qRT-PCR; **(D)** compared to control WB analysis for APOL3 was decreased in APOL3-Sg1 and APOL3-Sg2 cells. **Abbreviation:** WB western blotting; qRT-PCR Quantitative Real-time polymerase chain reaction.

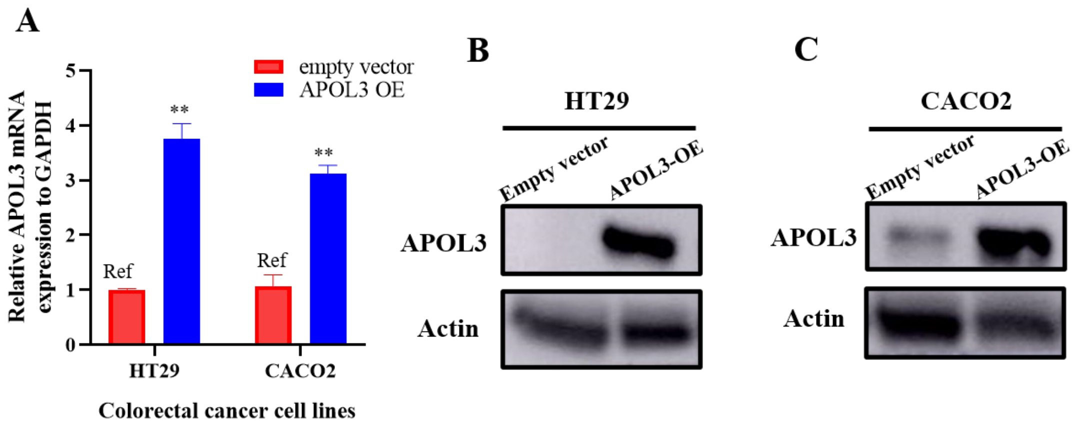

Figure S8: (A) Construction of APOL3-OE in HT29 and CACO2 cells and the relative APOL3 mRNA expression to GAPDH was analyzed by qRT-PCR; **(B)** compared to control WB analysis for APOL3 was decreased in APOL3-OE cells. **Abbreviation:** WB western blotting; qRT-PCR Quantitative Real-time polymerase chain reaction.

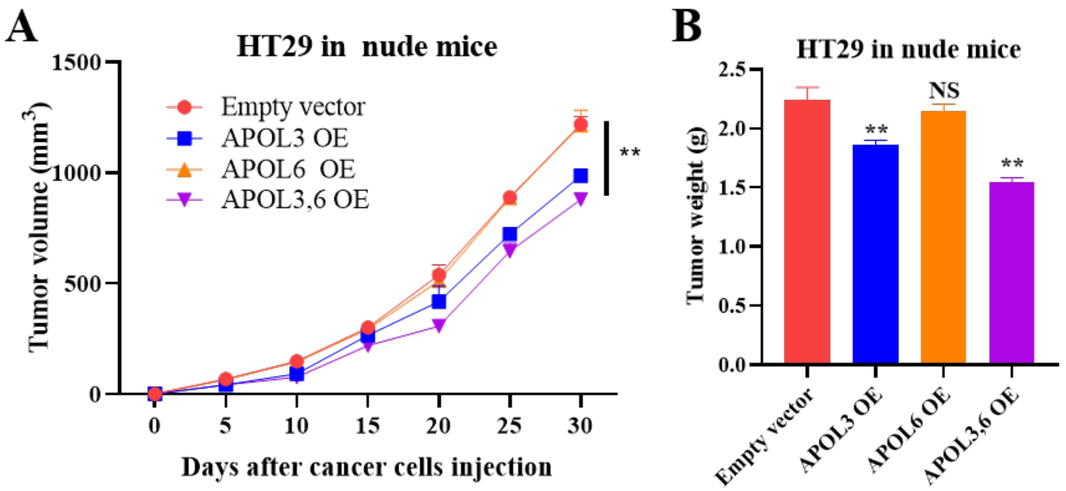

Figure S9: subcutaneous xenograft models of APOL3-overexpressed HT29 CRC cells were constructed. **(A)** tumor volume lines were drawn to compared the different cohorts overexpression of APOL3 significantly inhibits HT29 tumor growth in vivo; **(B)** tumor weight were also compared among different cohorts. **Abbreviation:** CRC colorectal cancer.

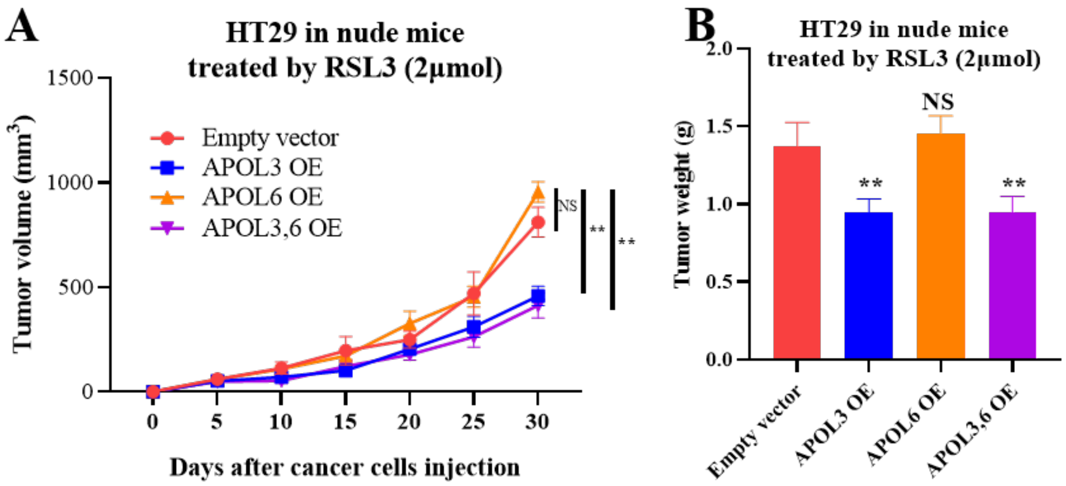

Figure S10: subcutaneous xenograft models of APOL3-overexpressed HT29 CRC cells were constructed and treated by RSL3. **(A)** tumor volume lines were drawn to compared the different cohorts overexpression of APOL3 significantly promoted HT29 cell death in vivo; **(B)** tumor weight were also compared among different cohorts. **Abbreviation:** CRC colorectal cancer.

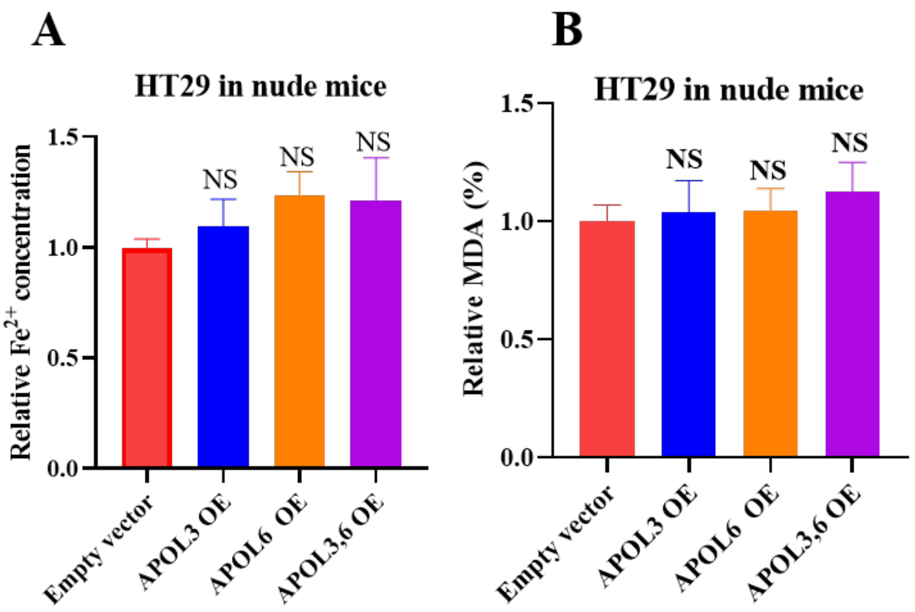

Figure S11: relative Fe2+ concentration **(A)** and MDA (%) **(B)** in subcutaneous xenograft models of HT29 cells.

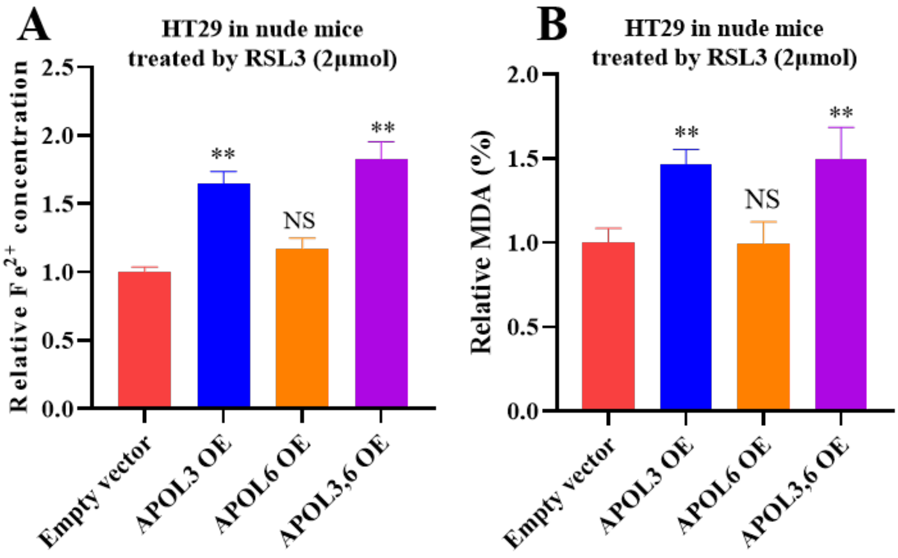

Figure S12: relative Fe2+ concentration **(A)** and MDA (%) **(B)** in subcutaneous xenograft models of HT29 cells treated by RSL3.

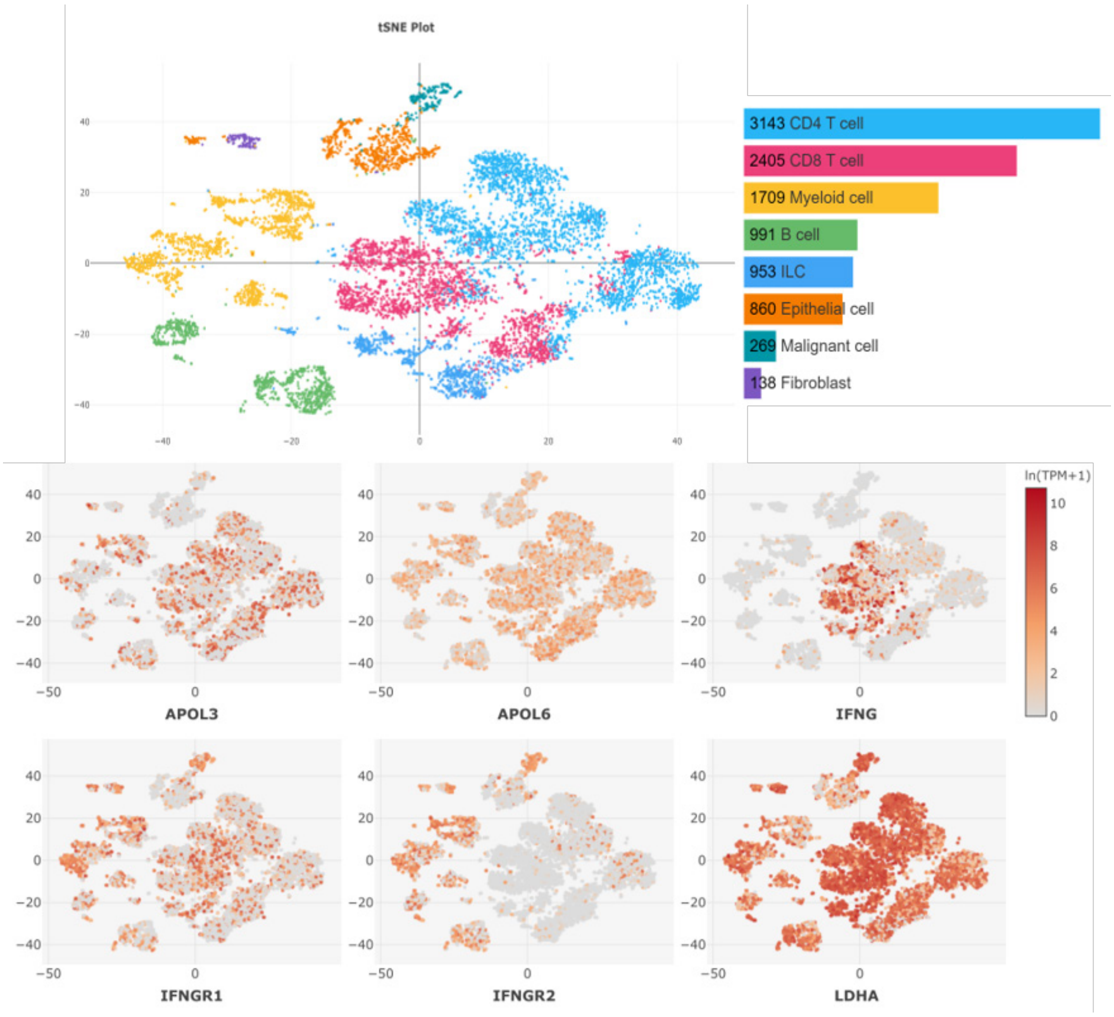

Figure S13: external single-cell RNA sequence validation for APOL3 on CRC microenvironment from public dataset (https://omnibrowser.abiosciences.com/). **Abbreviation:** CRC, colorectal cancer.
