# Supplementary material for: Apolipoprotein L3 enhances CD8+ T cell antitumor immunity of colorectal cancer by promoting LDHA-mediated ferroptosis
Source: Int J Biol Sci. 2023 Feb 13;19(4):1284–98. doi: 10.7150/ijbs.74985 (PMC10008698; doi:10.7150/ijbs.74985)
Supplement: Supplementary file 2 — Supplementary datasets. [file ijbsv19p1284s2.zip › datasetS7 EIC of Empty vector and APOL3-OE in RKO.docx]

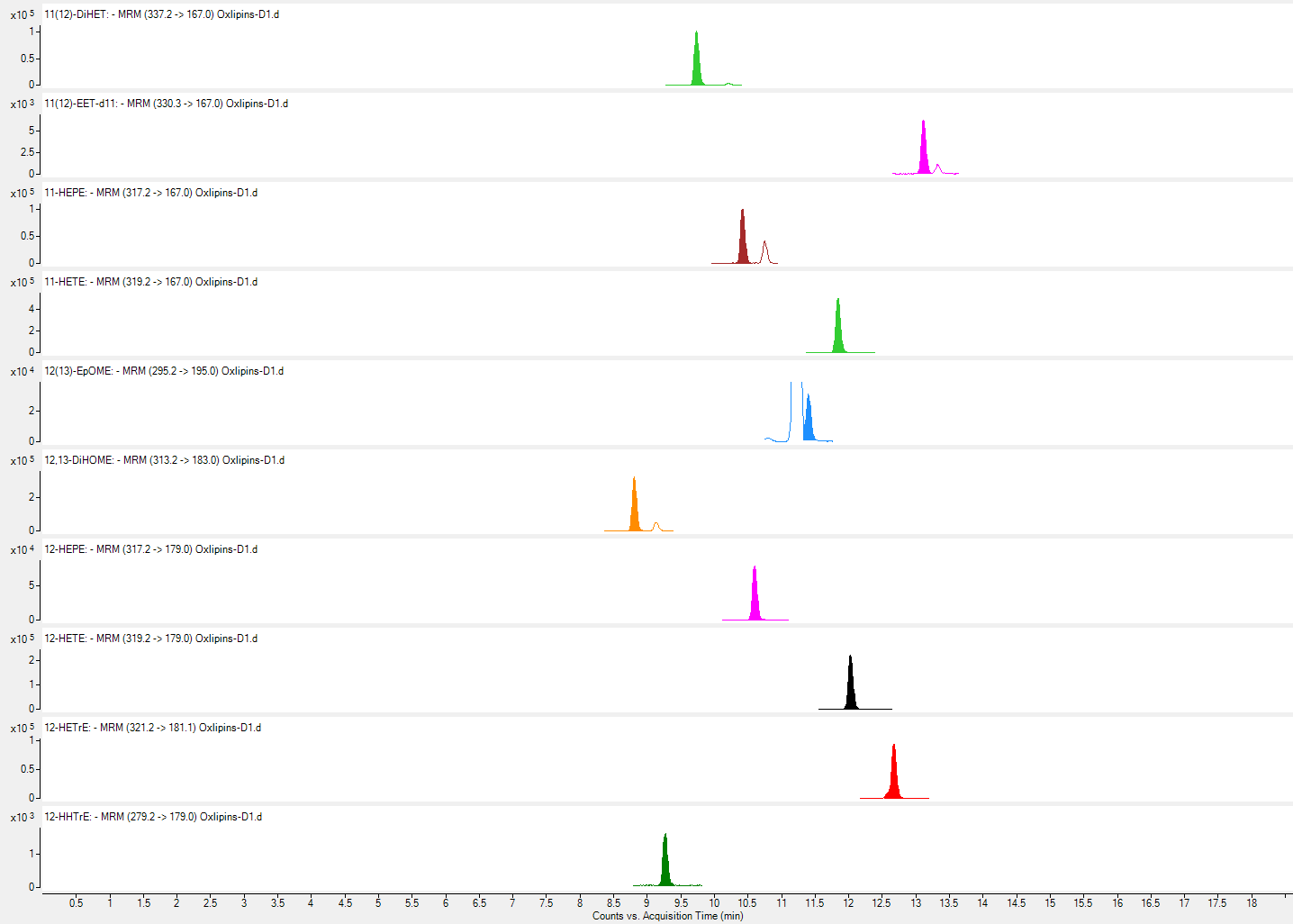

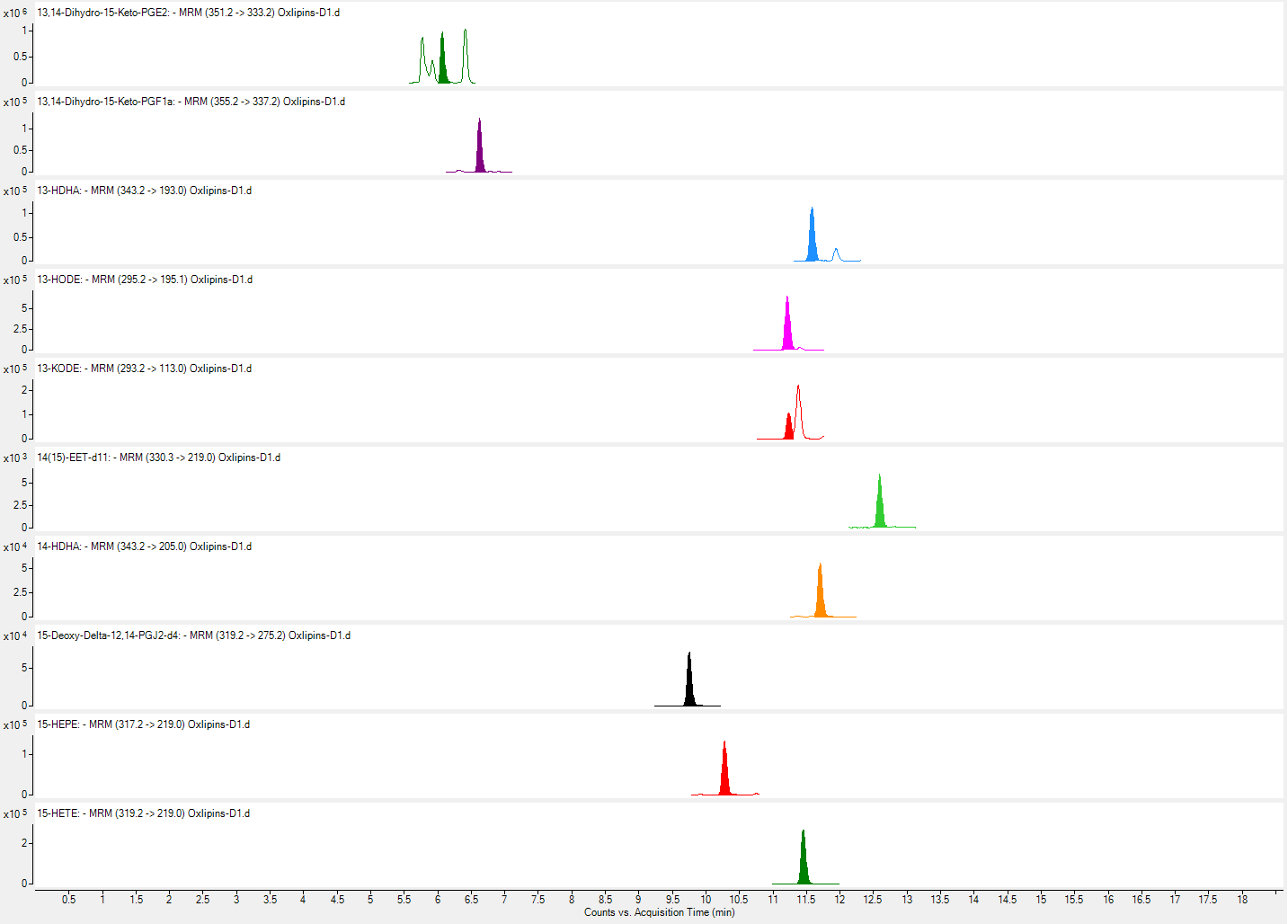

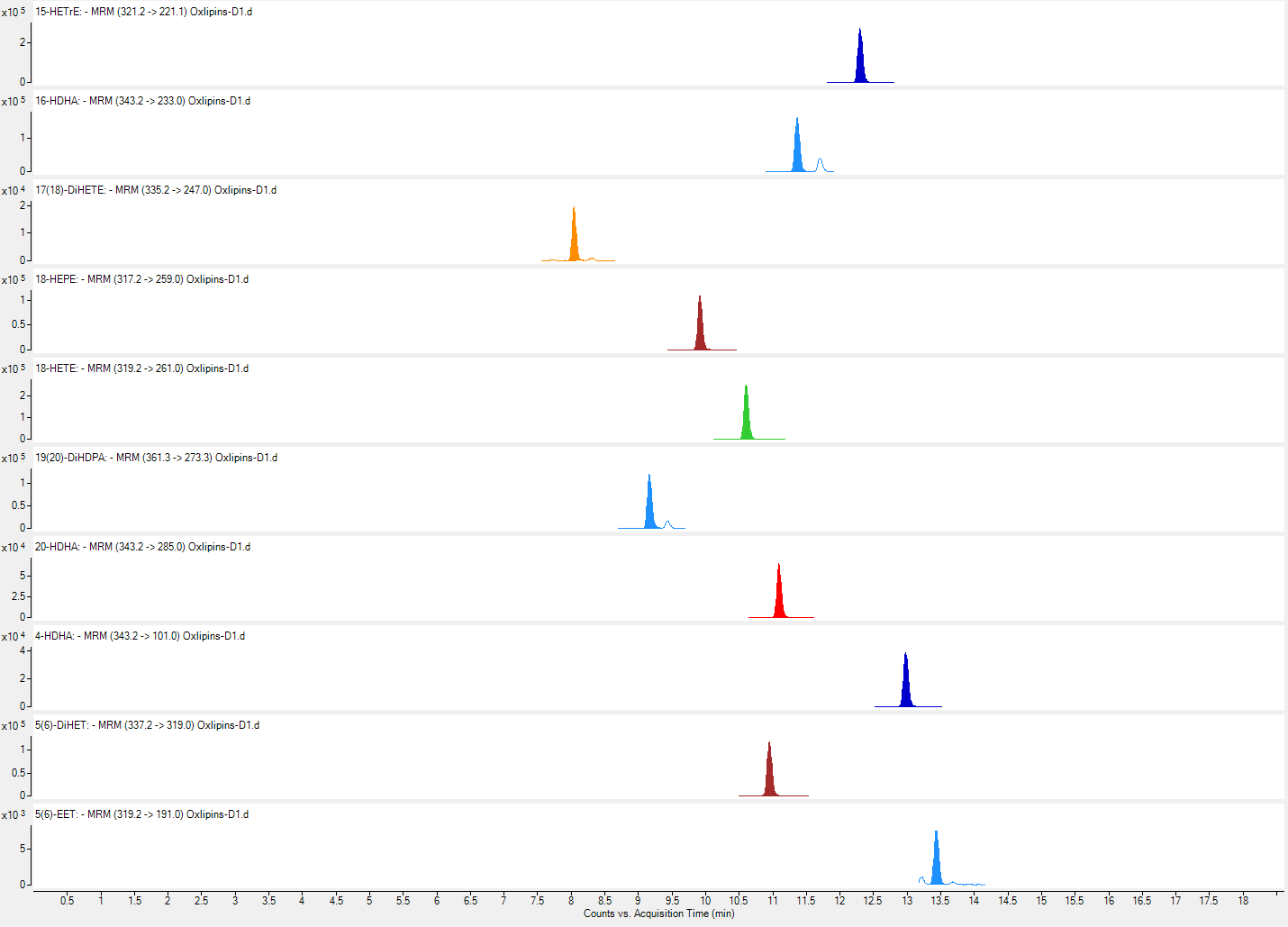

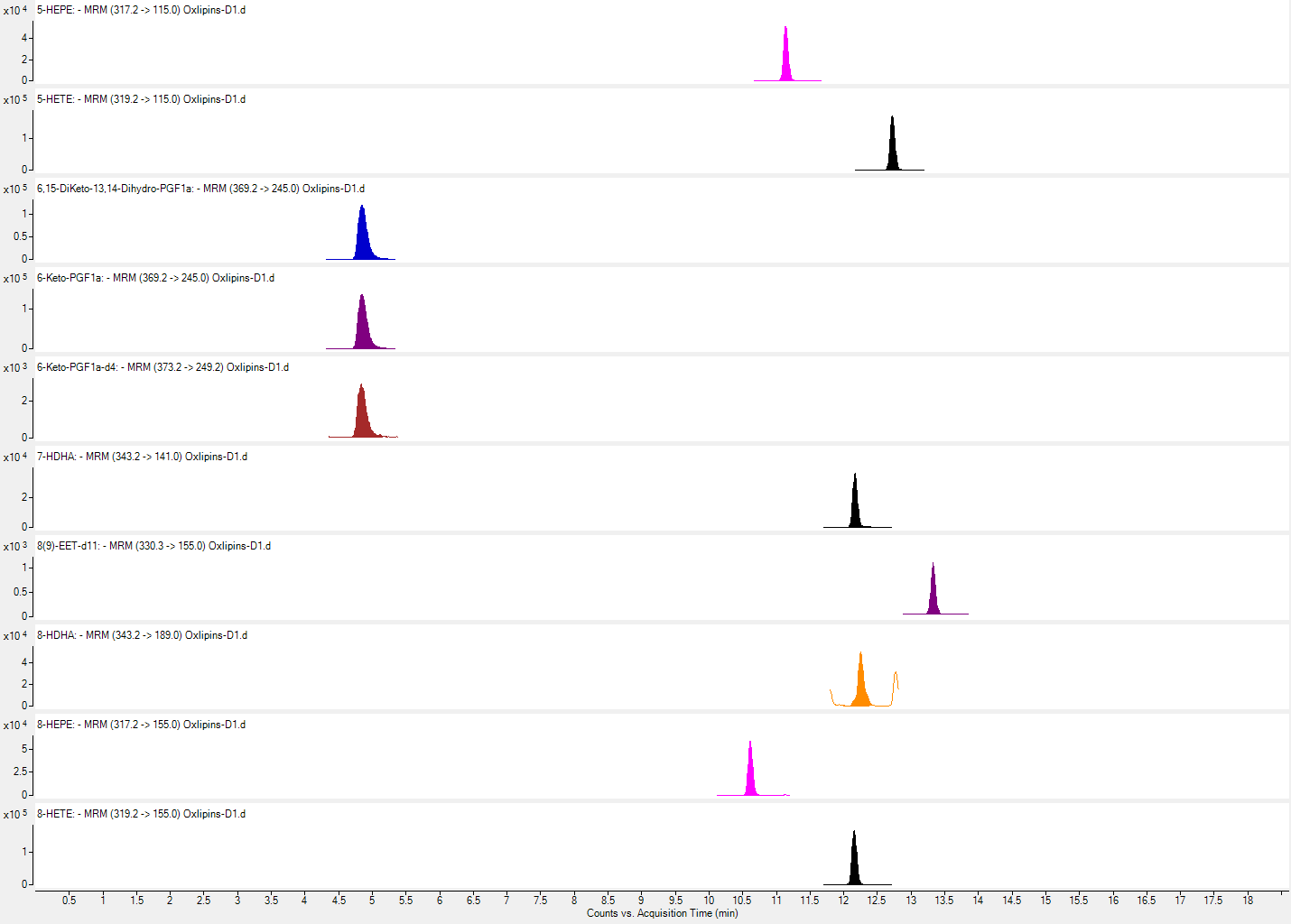

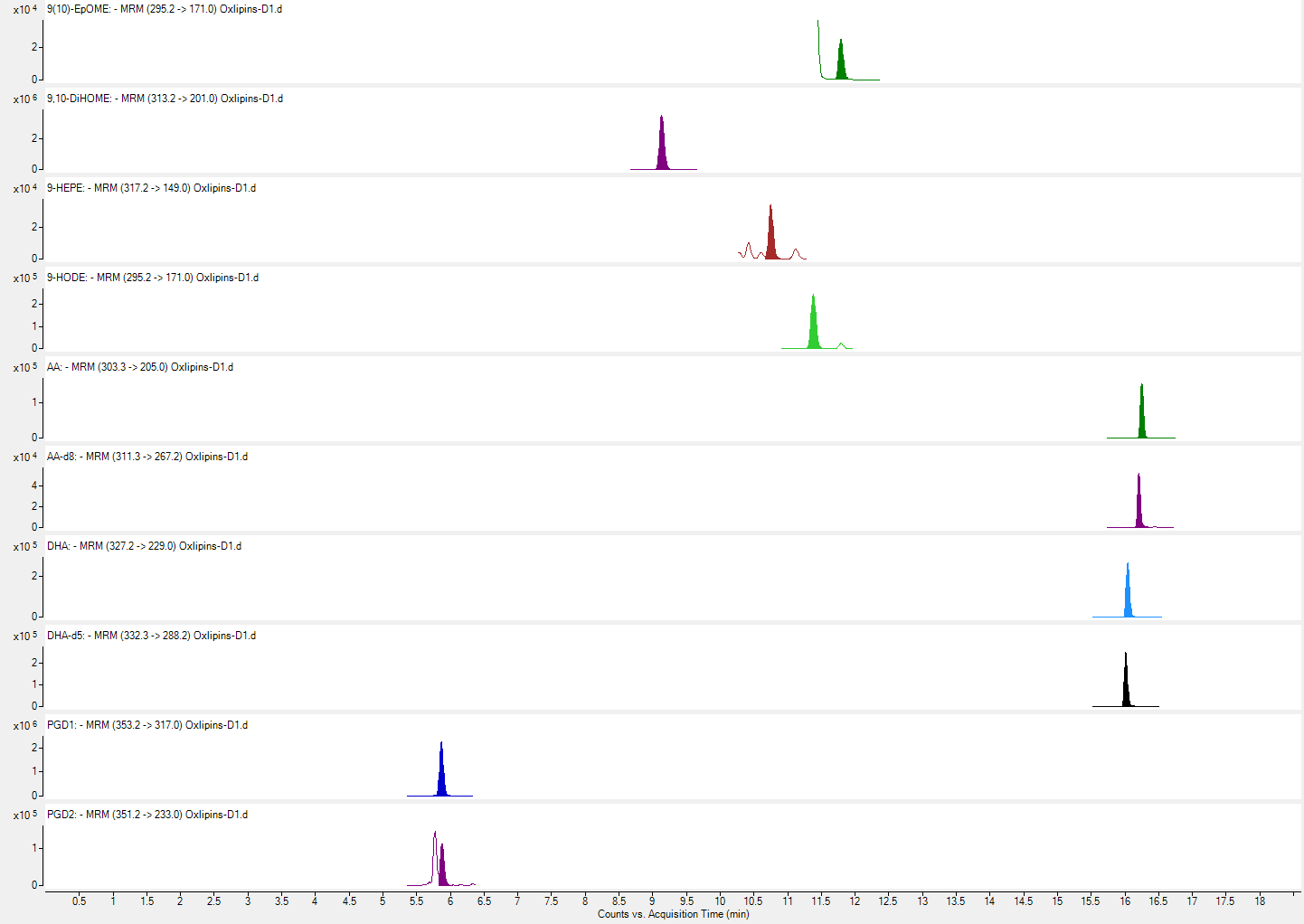

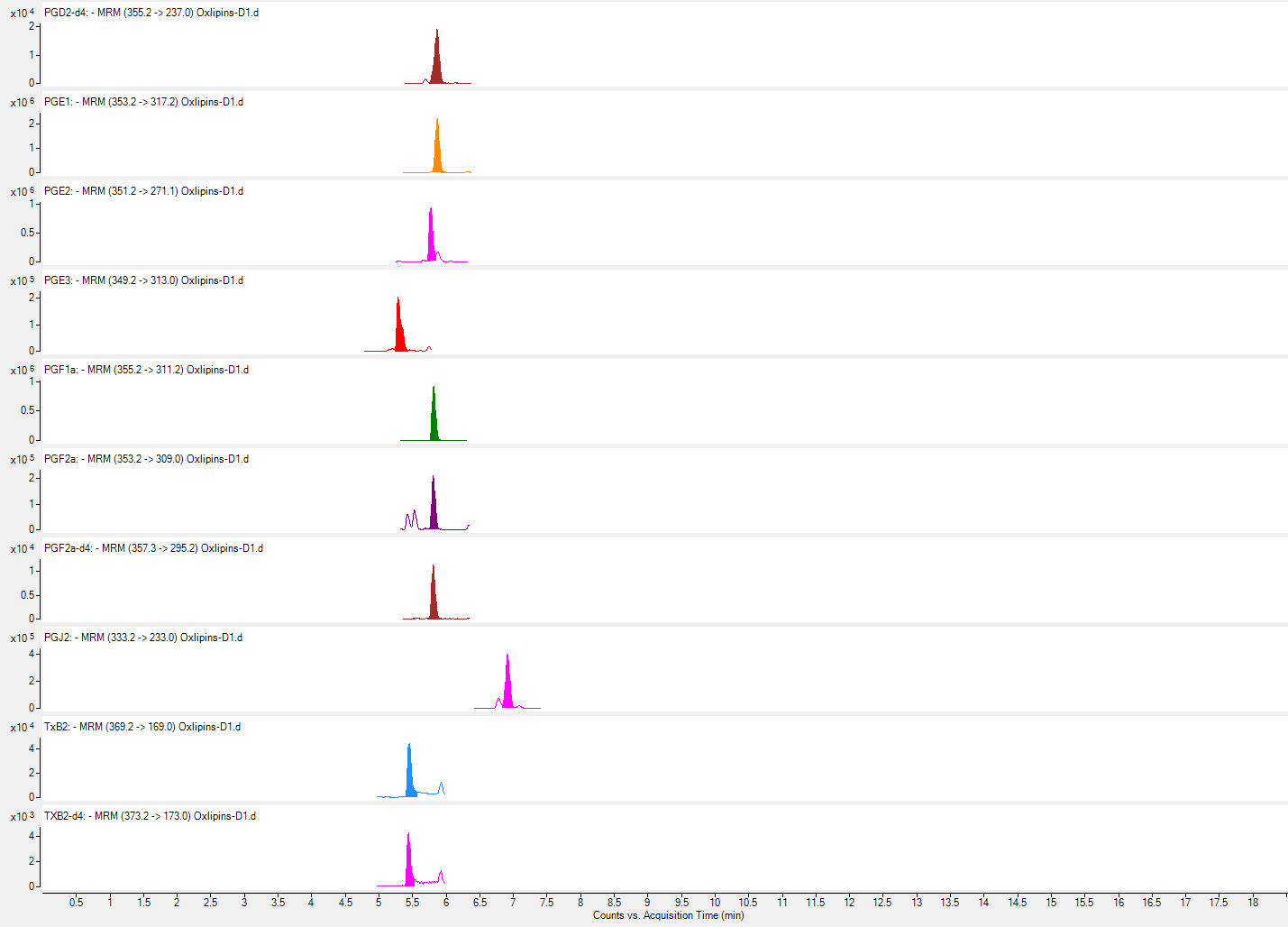


**Figure 1:** Extracted Ion Chromatogram (EIC) of RKO-empty vector cells


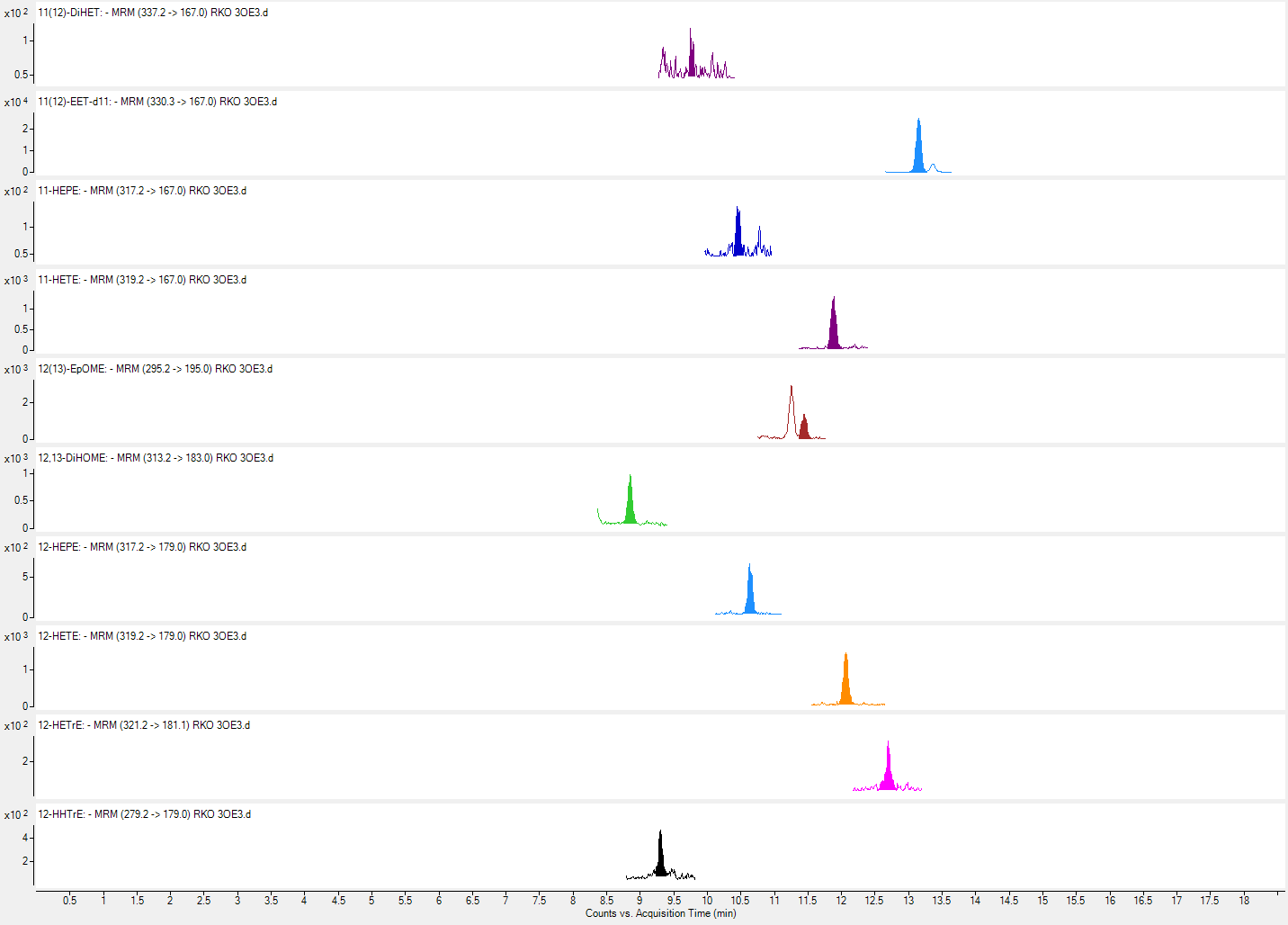

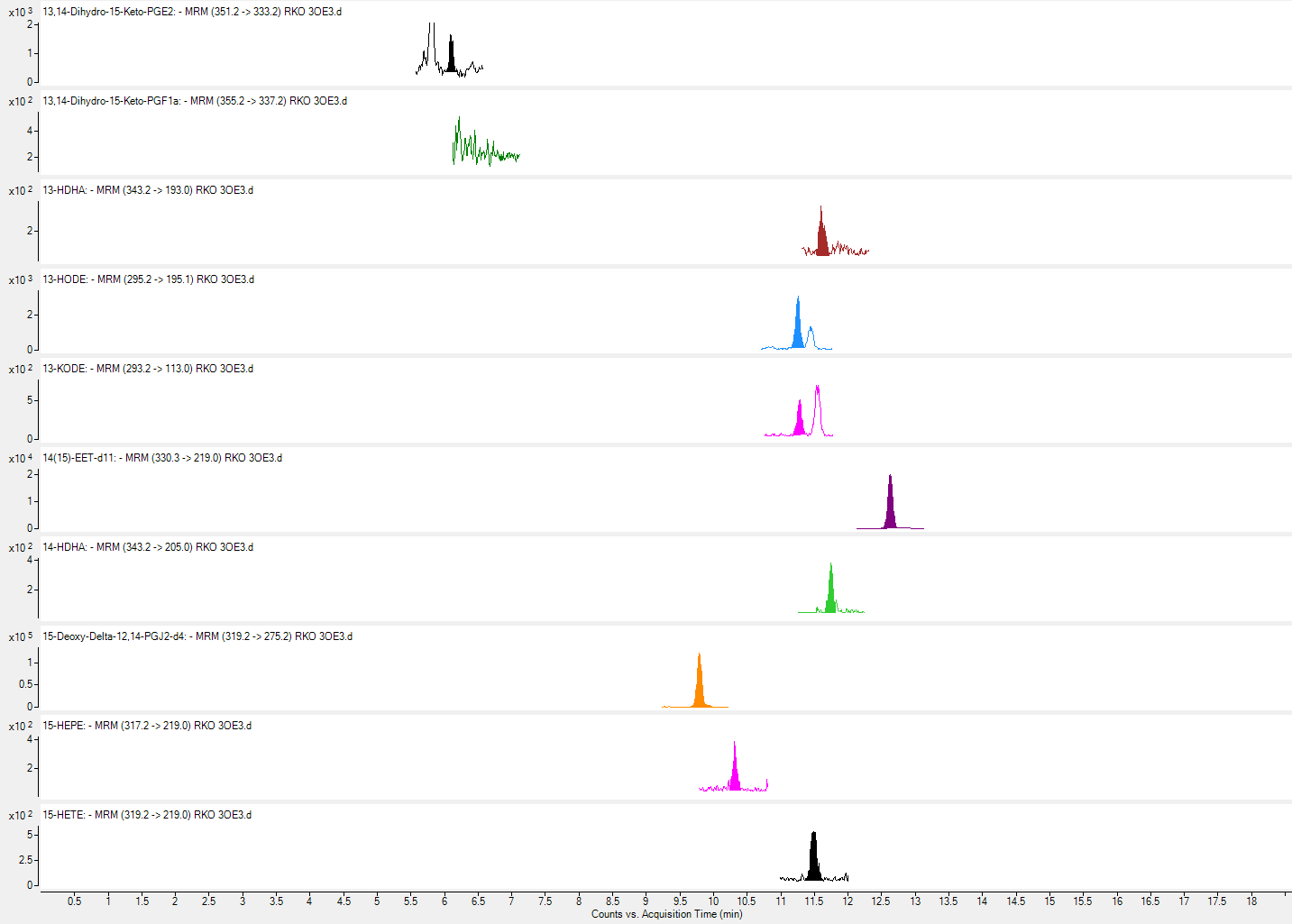

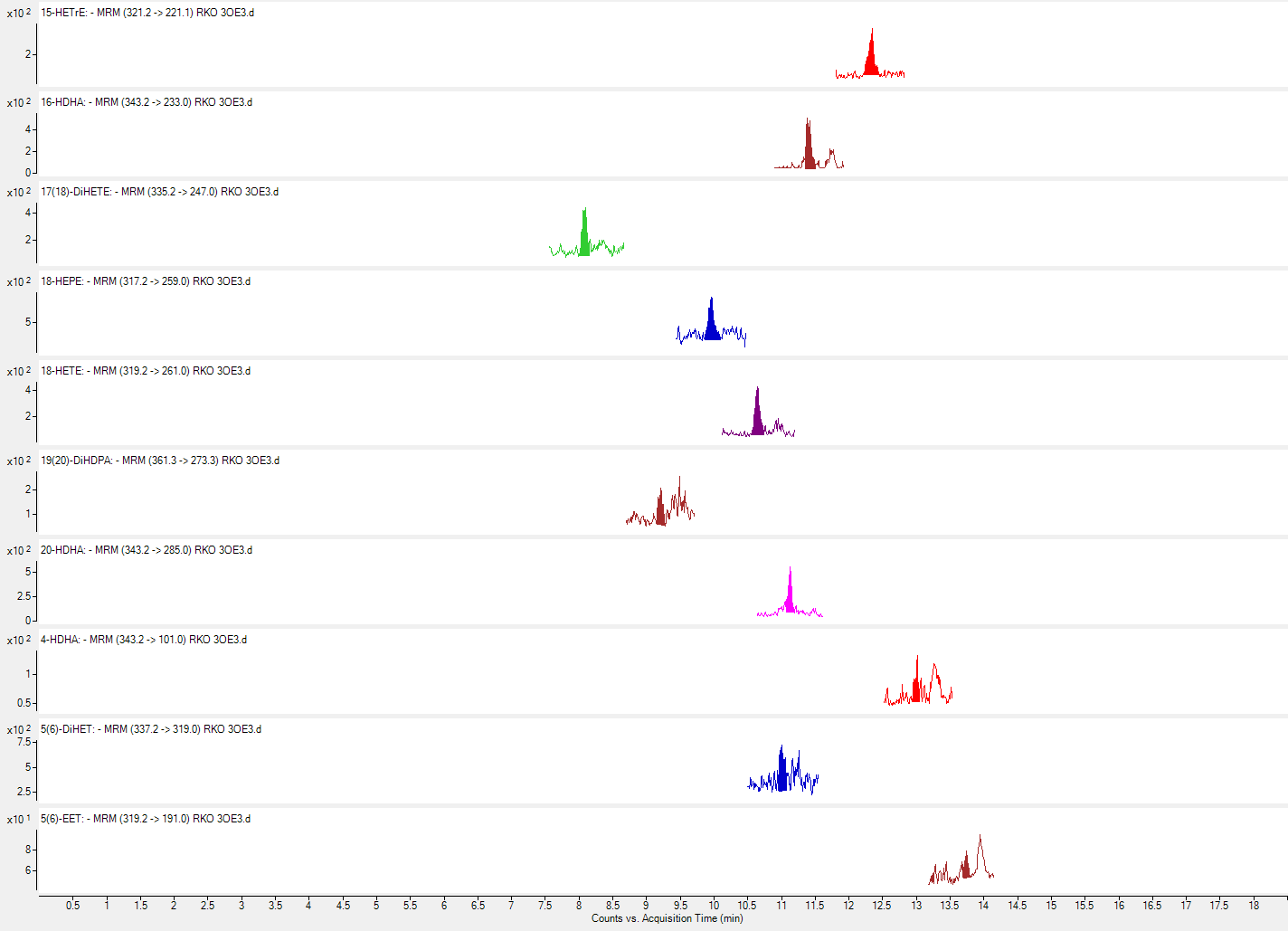

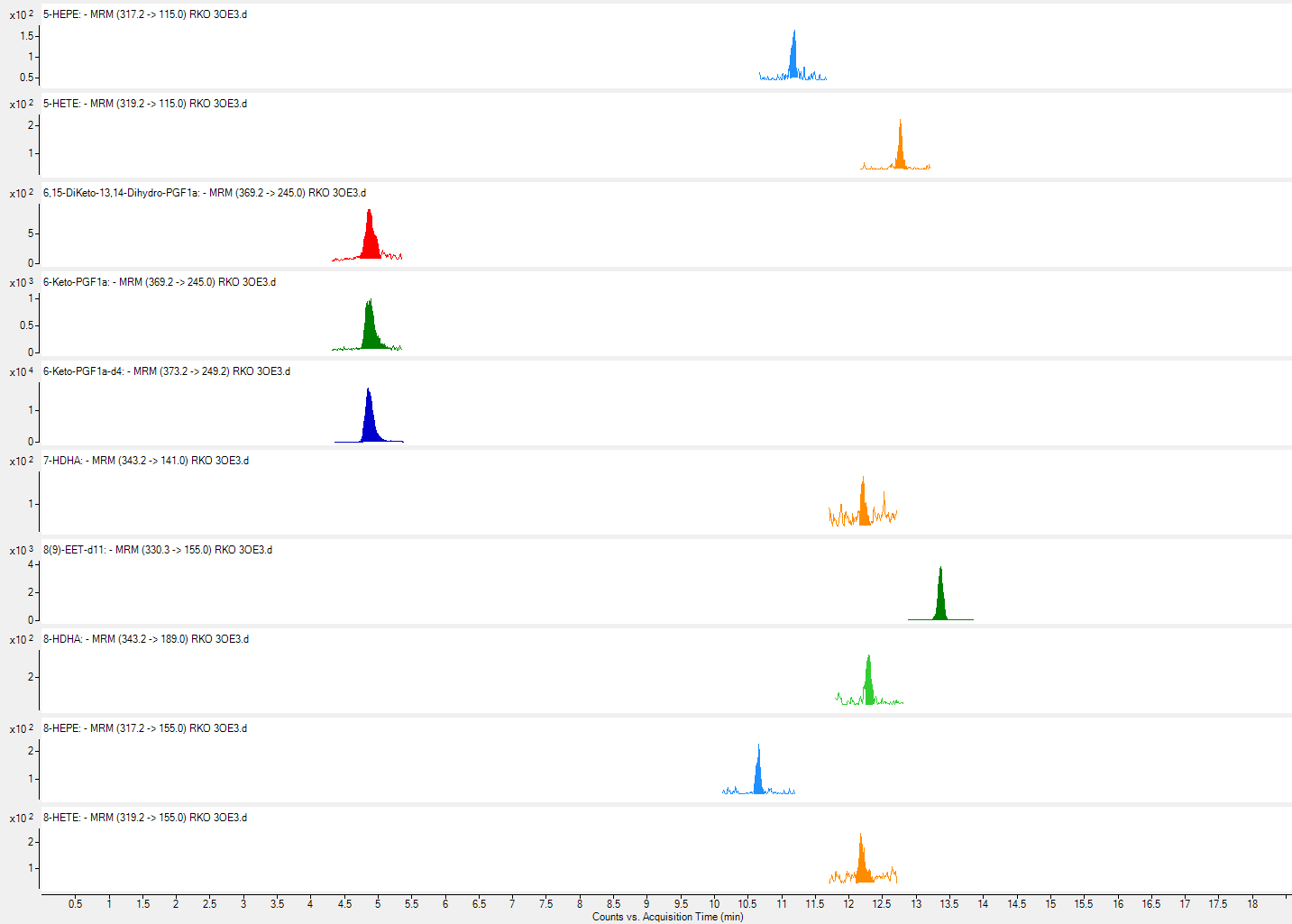

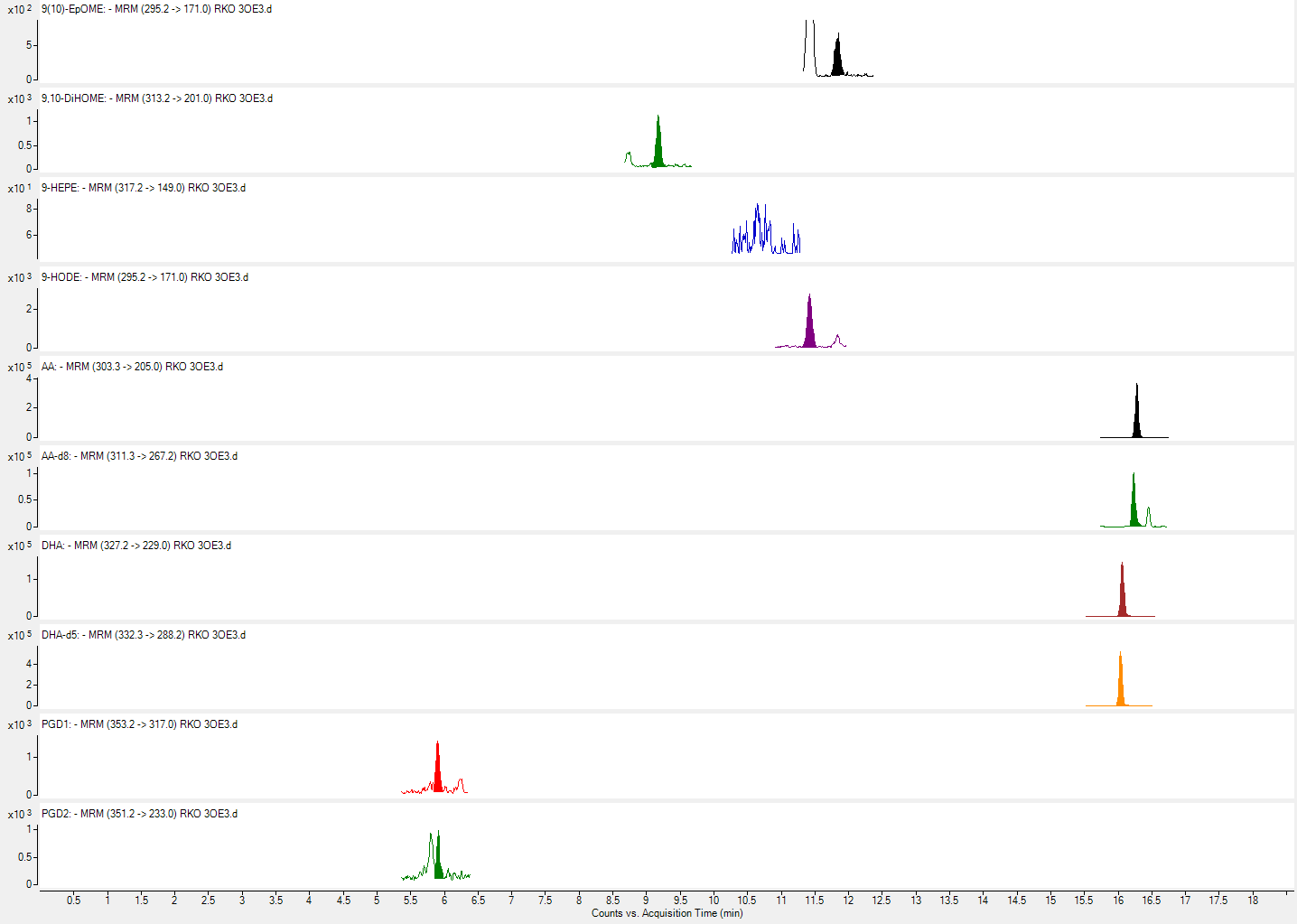

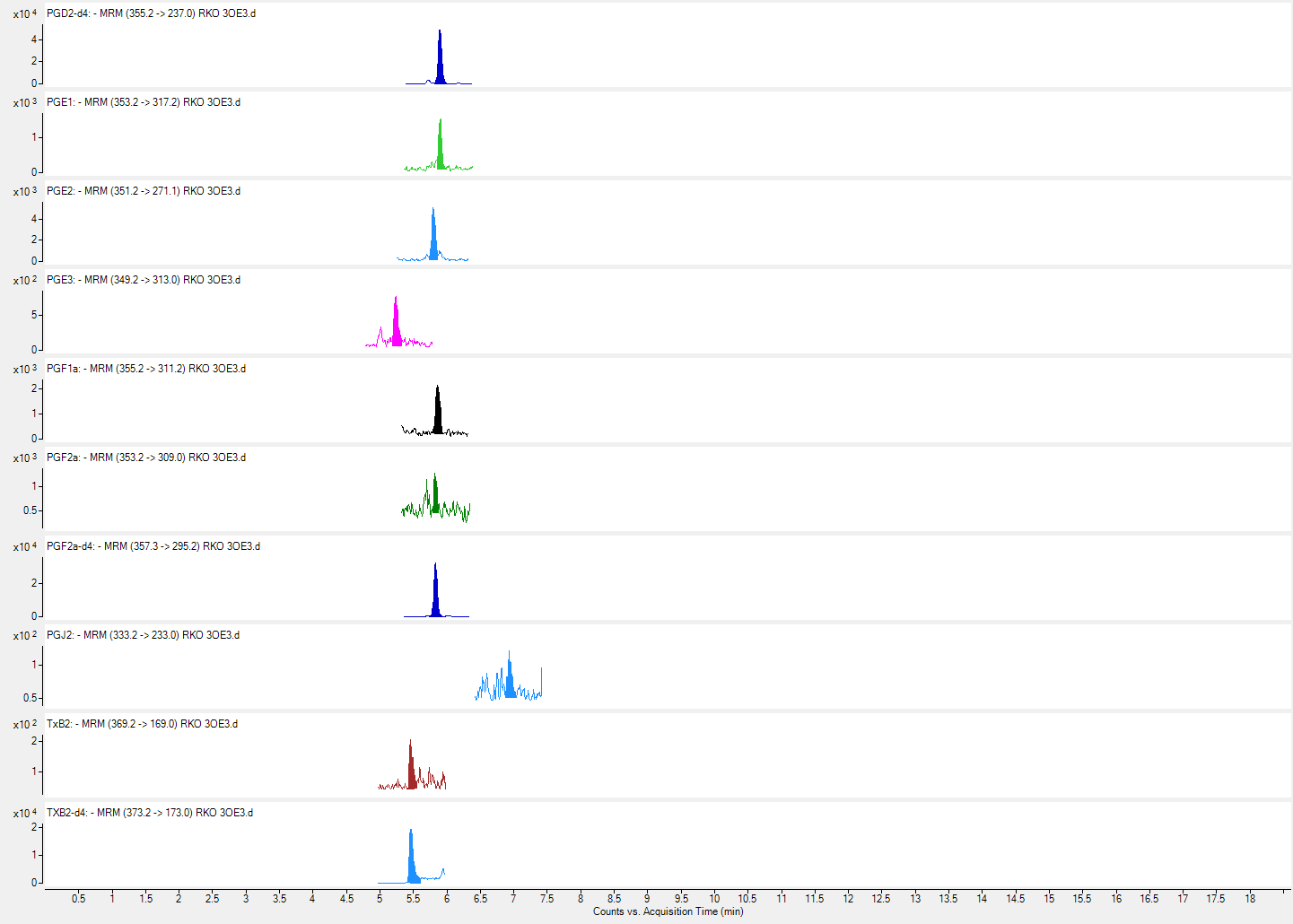


**Figure 2:** Extracted Ion Chromatogram (EIC) of RKO-APOL3-OE cells
